# Characterization of *Cellulomonas* sp. HM71 as potential probiotic strain for human health

**DOI:** 10.3389/fcimb.2022.1082674

**Published:** 2023-01-13

**Authors:** Monika Yadav, Tarun Kumar, Ranjeet Maurya, Rajesh Pandey, Nar Singh Chauhan

**Affiliations:** ^1^ Department of Biochemistry, Maharshi Dayanand University, Rohtak, Haryana, India; ^2^ INtegrative GENomics of HOst-PathogEn (INGEN-HOPE) Laboratory, Division of Immunology and Infectious Diseases, Council of Scientific & Industrial Research-Institute of Genomics and Integrative Biology (CSIR-IGIB), Delhi, India; ^3^ Academy of Scientific and Innovative Research (AcSIR), Ghaziabad, India

**Keywords:** human gut microbiota, probiotics, microbial augmentation, microbiome therapy, gluten metabolism, prolyl endopeptidase

## Abstract

*Cellulomonas* sp. HM71, a human gut microbe possesses metabolic machinery to catabolize antigenic gluten, hence, holds promises as microbial therapy to treat gluten-derived celiac disease. However, its efficacy, safety, and survivability in the gastrointestinal ecosystem await functional elucidation. The current study is designed to characterize *Cellulomonas* sp. HM71 for its physiological, genomic, and probiotic properties. The morphological and physiological assessment indicates it as a coccus-shaped gram-positive bacterium growing optimally at 30°C in a neutral environment (pH 7.0). *Cellulomonas* sp. HM71 showed continuous growth even in stressful environments (salinity up to 3% NaCl and 6% KCl), variable temperature (25°C to 35°C) and pH (5-9), antibiotics, and gastric and intestinal conditions. The *Cellulomonas* sp. HM71 genome harbors diversified genetic machinery to modulate humongous metabolic potential for the host. This was substantiated by the hemolytic and CaCo-2 cell line assay which confirms its cellular adherence and biosafety. Notably, genome analysis did not identify any pathogenic islands. Probiotic characterization indicates its potential to overcome waterborne infections and digestion-related disorders. Cumulatively, *Cellulomonas* sp. HM71 can be considered a probiotic strain for improving human health because of the highlighted functions.

## Introduction

The human gut is inhabited by a diverse array of microorganisms that holistically constitute the human gut microbiota (Yadav et al., 2018). The establishment and maturation of the human gut microbiota is an outcome of the complex yet dynamic host-microbe interactions (Yadav and Chauhan, 2022). The gut microbiome modulates several host metabolic and physiological processes. The human gut microbes are essential for human health as they have a characterized role in increasing biocatalytic efficiency, immunity, as well as pathogen defence of the human body (Yadav et al., 2018; Yadav and Chauhan, 2022). Though the gut microbiome is resilient, various factors such as dietary habits, age, and antibiotics could induce microbiome dysbiosis (Limbana et al., 2020). Microbiome dysbiosis could lead to the onset of various metabolic and physiological disorders, inclusive of autism, arthritis, obesity, and celiac disease.

Celiac disease affects approximately 1% of the global population (Jenab et al., 2020). It is triggered by ingesting gluten-containing foods in genetically susceptible individuals bearing human leukocyte antigens, HLA-DQ2, and HLA-DQ8. Gluten is a high molecular weight protein with proline and glutamine-rich repeat regions. Proline provides resistance from luminal and brush-border proteolytic degradation. Gastrointestinal enzymes like pepsin, trypsin, elastase 2A, elastase 3B, and carboxypeptidase A1 (CBPA1) catabolize dietry gluten (Gutiérrez et al., 2017). However, incomplete hydrolysis of gluten generates glutamine and proline-rich gliadin peptides (Roth et al., 2021). Tissue transglutaminase (TG2) deamidates these gliadin peptides to form negatively charged deamidated peptides (Roth et al., 2021). HLA-DQ2 and HLA-DQ8 molecules have a high affinity for deamidated gliadin peptides. The binding of deaminated peptides to HLA-DQ2 or HLA-DQ8 evokes a host immune response. Manifestations of autoimmune reaction are the destruction of the small intestinal mucosa and malabsorption of nutrients (Kumar et al., 2017). The auto-antigen (tissue transglutaminase 2) and dietary gluten are the other trigger factors for celiac disease (Kumar et al., 2017). Proline-specific proteolytic enzymes are essential for hydrolysis of proline-rich antigenic peptides; however, the host does not secrete any such enzyme in the gastrointestinal tract (Kumar et al., 2018). Recently, the limited activity of the prolyl endopeptidase was considered the causative agent for the onset of celiac disease (Singh et al., 2017). *Myxococcus xanthus*, *Flavobacterium menigosepticum*, *Sphingomonas capsulata*, *Rothia mucilaginosa*, *Rothia aeria*, *Lactobacilli*, *Aspergillus niger*, and lactic acid bacteria were characterized for the presence of prolyl endopeptidase activity (Lee et al., 2020; Zhu et al., 2022). The prolyl endopeptidase *of Myxococcus xanthus*, *Sphingomonas capsulata*, and *Flavobacterium meningosepticum* showed efficient hydrolysis of antigenic gliadin peptides and presented their therapeutic potential (Lee et al., 2020; Zhu et al., 2022). However, their suitability and efficacy in the host gastrointestinal tract limit their applicability to treat celiac diseases. Hereby, human gut microbes with gluten catabolic potential could overcome these issues. Recently, we isolated a human gut bacterium i.e. *Cellulomonas* sp. HM71 for antigenic gluten hydrolytic activity (Kumar et al., 2018). This microbe has the potential to overcome the onset of celiac disease; however, using gut microbes as potential probiotics avoids the safety concerns associated with using microbes as probiotics (Yadav et al., 2022). Several strains of *Cellulomonas* are already being employed as probiotics to improve the digestive health of animals compared to a diet without the additive (Lee et al., 2019), indicating the potential usage of *Cellulomonas* sp. HM71 as a probiotic for celiac disease management/cure. However, more extensive exploration/elucidation is required to decode the potential probiotic aspects of the human gut microbes. Hereby, the current study was designed to characterize *Cellulomonas* sp. HM71 for probiotic properties.

## Methods

### Morphological and phenotypic assessment of *Cellulomonas* sp. HM71

The morphological assessment of the human gut isolate *Cellulomonas* sp. HM71 was performed with the gram-staining kit (Himedia, cat No. K001-1KT). The growth pattern was analyzed after culturing 0.01 OD_600nm_ active cultures in Luria Bertani broth for 24 hrs with constant shaking at 200rpm at 37°C. Anaerobic growth of *Cellulomonas* sp. HM71 was observed after culturing 0.01 OD_600nm_ active cultures in an anaerobic basal broth (Himedia) in an anaerobic gas chamber. Carbohydrate utilization profile of *Cellulomonas* sp. HM71 was checked using the Hi-Carbo kit (Himedia, Cat No. KB009A-1KT, KB009B1-1KT, and KB009C-1KT).

### Assessment of *Cellulomonas* sp. HM71 genome

The genomic DNA of *Cellulomonas* sp. HM71 was isolated with the HiPurA™ Bacterial Genomic DNA Purification Kit (Himedia). Qualitative and quantitative assessment of the genomic DNA was performed with agarose gel electrophoresis and Qubit™ dsDNA HS assay (Invitrogen, USA), respectively. The genomic DNA of *Cellulomonas* sp. HM71 was sequenced on Illumina MiSeq using Nextera XT DNA Library Prep kit (Yadav et al., 2022). Quality filtering of raw reads, genome assembly, and genome completeness analysis was performed using standardized method (**Supplementary Method**) (Yadav et al., 2022). Evolutionary and phylogenetic relationships of the *Cellulomonas* sp. HM71 was checked with sequenced *Cellulomonas* genomes (**Supplementary Table S1**). Phylogenomic relationship of *Cellulomonas* sp. HM71 was assessed with other *Cellulomonas* genomes using M1CR0B1AL1Z3R webserver (https://microbializer.tau.ac.il/). The Average Nucleotide Identity and Tetra-correlation values were calculated using J-species software (http://jspecies.ribohost.com/jspeciesws/). The assembled genome was annotated with the PROKKA annotation pipeline (Seemann, 2014). PHASTER online tool was used for CRISPR identification and characterization (http://phaster.ca/). The pathogenic islands within the sequenced genome were detected with the Island Viewer 4 (https://www.pathogenomics.sfu.ca/islandviewer/resources/) following default parameters (https://www.pathogenomics.sfu.ca/islandviewer/about/). The dbCAN meta server (https://bcb.unl.edu/dbCAN2/blast.php) was used to identify CAZymes in the sequenced genome.

### Characterization of prolyl endopeptidase of *Cellulomonas* sp. HM71

Prolyl endopeptidase activity and gluten hydrolytic potential of *Cellulomonas* sp. HM71 was confirmed using a standardized methodology (Kumar et al., 2018) (**Supplementary Method SM2**). Annotated genome dataset was manually checked for the presence of the prolyl endopeptidase. Identified prolyl endopeptidase features were checked for the presence of conserved domains using CDD search (https://www.ncbi.nlm.nih.gov/Structure/cdd/wrpsb.cgi). Conserved functional residues were checked after aligning the prolyl endopeptidase (PEP) of *Cellulomonas* sp. HM71 with PEP sequences of *Myxococcus xanthus* and *Aeromonas punctata* using MUSCLE Alignment software (http://www.drive5.com/muscle). PEP sequence was checked for its topology, signal peptides, and phylogenetic homology (Kumar et al., 2018). Phylogenetic affiliation of Prolyl endopeptidase (PEP) was performed with S9 Serine protease family members using the Neighbor-Joining (NJ) method of phylogenetics of MEGA-X software (https://www.megasoftware.net/).

### Physiological assessment of *Cellulomonas* sp. HM71 in a stressful environment

Growth of *Cellulomonas* sp. HM71 was observed in gastrointestinal conditions (gastric, intestinal, bile salts and lysozyme) and in the presence of metal/metalloids, and salts (Yadav et al., 2022). Active bacterial culture of 0.05 OD (600nm) was used with an interval of 2hrs in LB broth. Antibiotic susceptibility of Human gut isolate HM71 was observed against antibiotic discs of Amikacin, Amoxicillin, Bacitracin, Cephalothin, Erythromycin, Novobiocin, Oxytetracycline, Vancomycin, Ceflnaxone, Ceftazidime, Cefotaxime, Lincomycin, Netilin, and Ofloxacin (Himedia, Cat No. OD034R-1PK and OD003R-1PK) (Yadav et al., 2022). Cell surface hydrophobicity and auto-aggregation tendency of the *Cellulomonas* sp. HM71 was also observed (Yadav et al., 2022).

### Bio-safety evaluation of the *Cellulomonas* sp. HM71

Hemolytic activity and cellular toxicity of *Cellulomonas* sp. HM71 was assessed using the blood agar plate (5% v/v) and the Caco-2 cell line (**Supplementary Method SM3**), respectively (Yadav et al., 2022). Cellular adherence of the isolate *Cellulomonas* sp. HM71 was also analyzed (**Supplementary Method SM3**) using Caco-2 cell line (Yadav et al., 2022).

### Assessment of the health maintenance properties of *Cellulomonas* sp. HM71

The antimicrobial nature of the *Cellulomonas* sp. HM71 was assessed against the pathogens *Staphylococcus aureus* (MTCC No. 96)*, E.coli* (MTCC No. 443), and *Salmonella typhi* (MTCC No. 98). Co-aggregation tendency of the *Cellulomonas* sp. HM71 with pathogens (*Staphylococcus aureus* (MTCC No. 96)*, E.coli* (MTCC No. 443), and *Salmonella typhi* (MTCC No. 98)) was also assessed (Yadav et al., 2022). Human gut isolate HM71 was assessed for activity towards anti-amylase, anti-lipase, cholesterol removal, and bile salt hydrolysis (Yadav et al., 2022). It was screened for the activity of lactase, laccase, peroxidase, and phosphatase (Yadav et al., 2022).

## Results

### Morphological and physiological characterization of *Cellulomonas* sp. HM71

**Figure 1:**
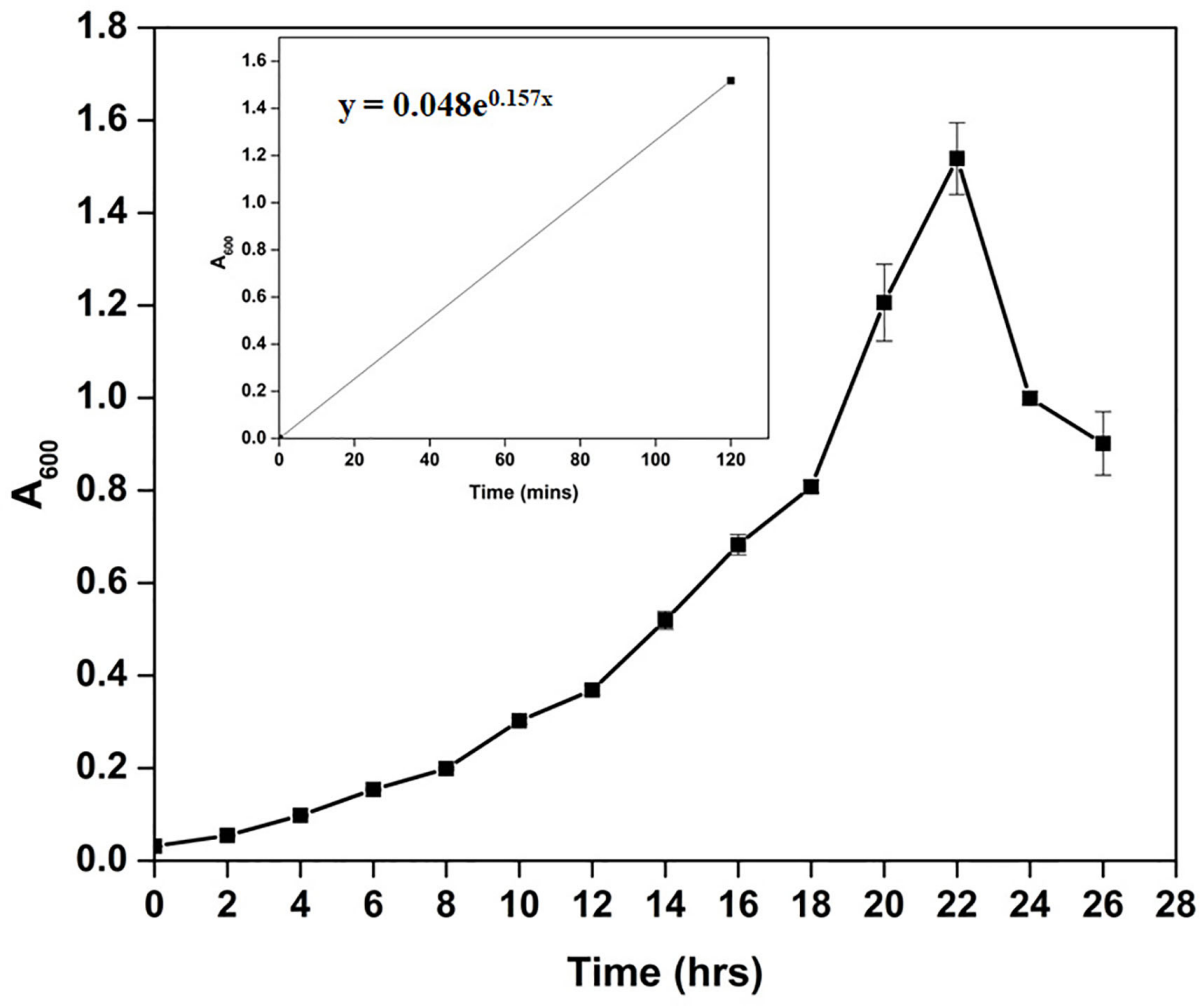
Growth pattern analysis of the bacterial isolate strain *Cellulomonas* sp. HM71 in Luria-Bertani broth for 24hrs at 37°C with constant shaking at 200 rpm. Each point in the graph is the mean value of readings observed in triplicate experiments.

Microscopic observation of *Cellulomonas* sp. HM71 highlighted it as coccus-shaped gram-negative bacteria. Growth pattern analysis indicated that it attains maximum growth within 26 hrs (**Figure 1**) with a doubling time of 264.897 mins in aerobic growth conditions. It also showed a growth (0.508 OD at 600nm) after incubating the culture for 24 hrs at 37°C in the anaerobic growth conditions. Substrate utilization assay of *Cellulomonas* sp. HM71 indicated its potential to utilize Lactose, Xylose, Maltose, Fructose, Dextrose, Galactose, Trehalose, Sucrose, L-arabinose, Mannose, Inulin, Glycerol, Salicin, Rhamnose, Cellobiose, Melezitose, Esculin, and D-arabinose, out of the given 35 substrates (**Table 1**; **Supplementary Table S2**). Substrate utilization preference was similar to the other species of *Cellulomonas* (**Supplementary Table S2**).

**Table 1:** Physiological, morphological, and biochemical characterization of *Cellulomonas* sp. HM71.

Property	Term
**Gram stain**	Positive
**Cell shape**	Coccus
**Temperature range**	25^0^ to 35°C
**Optimum temperature**	30°C
**pH range**	5-9
**Optimum pH**	7.0
**Habitat**	Human gut
**Salinity/Metal/Metalloid resistance**	Up to 3% NaCl and 6% KCl; Silver nitrate (up to 1mM); Cadmium chloride (up to 0.5mM); Lead acetate (up to 2mM); Sodium arsenate (up to 25mM); Potassium dichromate (up to 2mM)
**Substrate utilization preference** **Oxygen requirement**	Lactose, Xylose, Maltose, Fructose, Dextrose, Galactose, Trehalose, Sucrose, L-arabinose, Mannose, Inulin, Glycerol, Salicin, Rhamnose, Cellobiose, Melezitose, Esculin, D-arabinose Aerobic
**Biotic relationship**	Host-associated
**Pathogenicity**	Non-pathogenic

### Genomic characterization of *Cellulomonas* sp. HM71

**Figure 2:**
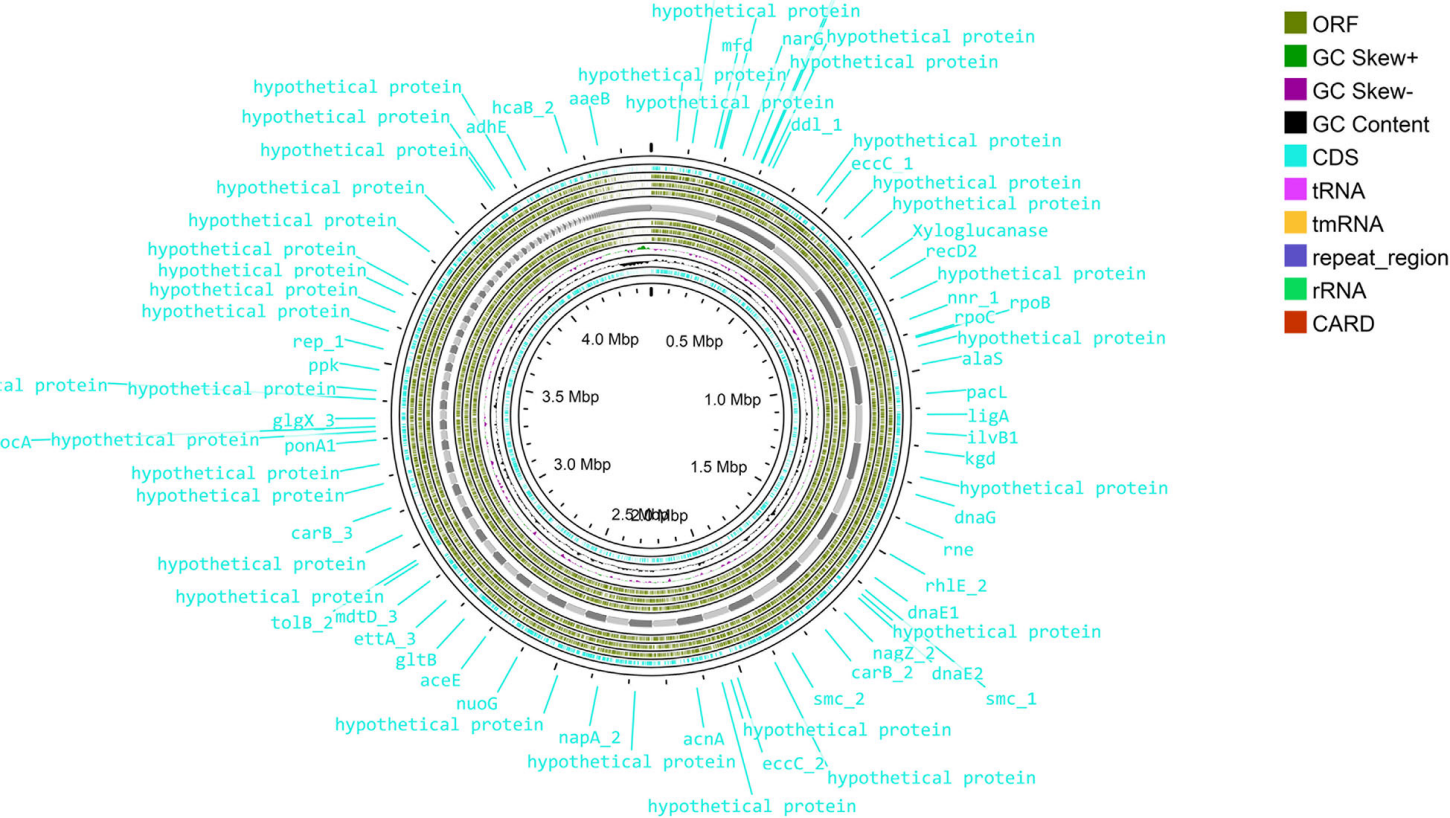
Genome map of the *Cellulomonas* sp. HM71. The circular genome map was drawn using Proksee online tool (https://proksee.ca/) that uses a complete genome sequence and annotates it using PROKKA. It also identifies resistance genes using CARD identifier.

Sequence assembly of *Cellulomonas* sp. HM71 highlighted 239 contigs amounting to 3,643,765 base pairs with 62.14% GC content (**Supplementary Table S3**). A quantitative assessment of the completeness in terms of the expected gene content of a genome assembly or annotated gene set (https://busco.ezlab.org/) was done. The BUSCO assessment resulted in 100% genome assembly and 124 complete, 124 single copies, 0 duplicated copies, 0 fragmented, and 0 missing conserved proteins within the bacterial genome (**Supplementary Table S3**). Genome annotation identified 3,996 coding genes and 53 tRNAs and 3 rRNAs in the genome of *Cellulomonas* sp. HM71 (**Figure 2** **;** **Table 2**). Genome characterization identified 34 features associated with antibiotic resistance, 30 protein features associated with oxidative stress tolerance, and 6 features associated with heat tolerance (**Supplementary Table S4**). A number of antibiotic and metal/metalloid resistance gene clusters were also observed in its genome (**Supplementary Tables S4**, **S5**). Additionally, CAZymes annotation with HMMER resulted in a total of 30 CAZymes clusters (**Supplementary Table S6**). No pathogenic islands/genes were determined within the genome of the microbial isolate. Additionally, the virulence genes were manually searched within the genome of the microbial isolate resulting in the absence of any genes relating to the pathogenic behavior of the isolated microbe.

**Table 2:** PROKKA annotated genome features of *Cellulomonas* sp. HM71.

Genome feature	Value
**Bases**	4480860
**Contigs**	412
**CDS**	3966
**Repeat region**	1
**tmRNA**	1
**rRNA**	3
**tRNA**	53

### Genome comparison of *Cellulomonas* sp. HM71

**Figure 3:**
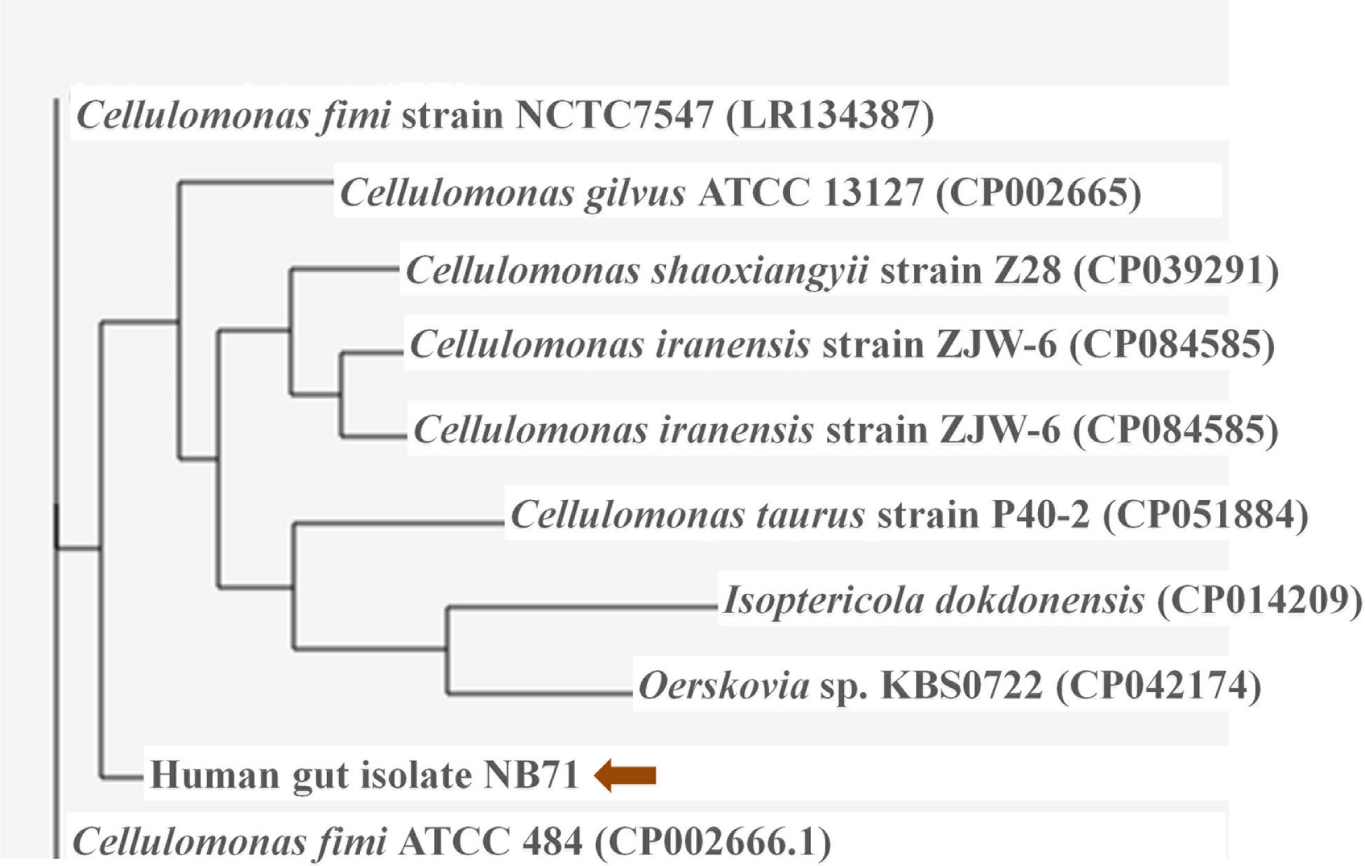
Phylogenetic assessment of the genome of *Cellulomonas* sp. HM71 with the genomes of *Cellulomonas* species. The phylogenetic relationship of the gut bacterial isolate strain HM71 was assessed with other *Cellulomonas* genomes using M1CR0B1AL1Z3R webserver (https://microbializer.tau.ac.il/). The online tool extracts ORFs, detects OGs, extracts OG sequences, infers a core proteome, and reconstructs the species phylogeny. The tree was drawn by taking Maximal e-value cutoff: 0.01; Identity minimal percent cutoff: 80.0%; Minimal percentage for core: 100.0% with no bootsrapping.

The in-house sequenced genome of *Cellulomonas* sp. HM71 was compared with the genomes of the *Cellulomonas* species (**Supplementary Table S1**) to elucidate genome-level similarities and variations. The comparison was made towards the genome size, coding sequences, tRNA, and the rRNA (**Supplementary Table S1**). The Average Nucleotide Identity (ANI) value among all the *Cellulomonas* species was ∼66-97%, which is towards the lower end of the 62–100% spectrum of interspecies variation within a genus (Bryson et al., 2009), suggesting substantial genomic diversity. This observation was reaffirmed by tetra correlation among member species, highlighted by a wide distribution of z-scores (**Supplementary Table S7**). The isolated gut microbe shared high ANI (84.51%) with *Cellulomonas fimi* ATCC 484 while ANIs with *Cellulomonas flavigena* DSM 20109, *Cellulomonas gilvus* ATCC 13127, *Cellulomonas iranensis* strain ZJW-6, *Cellulomonas shaoxiangyii* strain Z28, and*Cellulomonas taurus* strain P40-2were found to be 77.31%, 77.89%, 77.91%, 77.60%, and 74.69% respectively (**Table 3** **)**. A z-score value of 0.98105 during tetra-correlation scoring corroborates its similarity with *Cellulomonas fimi* ATCC 484. Other *Cellulomonas* strains also shared higher similarities with the gut isolate (z-score ~0.90-0.99) (**Supplementary Table S7**). Phylogenetic analysis of *Cellulomonas* sp. HM71 indicated its similarity with *Cellulomonas* (**Figure 3**). *Cellulomonas* sp. genome had a total of 4758 Cluster of Orthologous genes (COGs). The distribution of COGs varies across the microbial group (**Supplementary Figure S1**). These results indicate genome plasticity within a microbial species, which could be attributed to niche-specific genome evolution (Woodcock et al., 2017).

**Table 3:** Average Nucleotide Identity (ANI) of *Cellulomonas* sp. HM71 with reference to other *Cellulomonas* species.

ANIB (Aligned nucleotides %)	Human gut isolate HM71	*Cellulomonas fimi* ATCC 484	*Cellulomonas flavigena* DSM 20109	*Cellulomonas fimi* strain NCTC754	*Cellulomonas gilvus* ATCC 13127	*Cellulomonas iranensis* strain ZJW-6	*Cellulomonas shaoxiangyii* strain Z28	*Cellulomonas taurus* strain P40-2	*Isoptericola dokdonensis* DS-3	*Oerskovia* sp. KBS0722
**Human gut isolate HM71**	*	84.51 (59.78)	77.31 (39.76)	84.48 (58.89)	77.89 (39.38)	77.91 (40.25)	77.60 (40.52)	74.69 (30.56)	73.93 (30.46)	74.91 (29.40)
***Cellulomonas fimi* ATCC 484**	84.63 (61.78)	*	77.10 (41.72)	99.89 (90.95)	77.82 (41.91)	77.74 (43.01)	77.64 (42.12)	74.66 (31.89)	73.64 (31.25)	74.58 (30.37)
***Cellulomonas flavigena* DSM 20109**	77.60 (43.10)	77.46 (43.61)	*	77.42 (43.14)	76.74 (39.17)	82.42 (51.65)	79.78 (47.85)	74.65 (30.78)	73.74 (30.82)	74.38 (29.39)
***Cellulomonas fimi* strain NCTC754**	84.68 (61.72)	99.99 (92.43)	77.08 (41.76)	*	77.81 (42.04)	77.81 (42.96)	77.71 (42.18)	74.63 (32.39)	73.71 (31.33)	74.57 (30.38)
***Cellulomonas gilvus* ATCC 13127**	78.16 (48.77)	78.11 (50.38)	77.01 (44.43)	78.11 (49.77)	*	77.51 (46.21)	76.94 (42.76)	74.68 (36.56)	73.48 (33.76)	74.47 (32.89)
***Cellulomonas iranensis* strain ZJW-6**	78.75 (39.83)	78.54 (40.99)	82.87 (47.74)	78.51 (40.58)	77.87 (37.12)	*	81.00 (44.20)	75.25 (31.11)	74.55 (28.88)	74.99 (29.80)
***Cellulomonas shaoxiangyii* strain Z28**	78.07 (43.83)	78.06 (43.85)	80.09 (47.35)	78.04 *(43.39)*	76.99 (37.42)	80.76 (47.75)	*	74.88 (31.90)	74.05 (31.18)	74.67 (29.31)
***Cellulomonas taurus* strain P40-2**	74.53 (42.09)	74.55 (42.59)	74.17 (39.18)	74.53 (42.09)	74.35 (40.81)	74.13 (44.55)	74.35 (39.51)	*	72.60 (34.37)	73.10 (36.16)
***Isoptericola dokdonensis* DS-3**	73.95 (35.27)	73.92 (34.68)	73.59 (32.68)	73.92 (34.32)	73.41 (31.60)	73.80 (33.52)	73.78 (32.91)	73.08 (28.75)	*	74.76 (36.07)
***Oerskovia* sp. KBS0722**	74.47 (32.56)	74.28 (32.59)	73.73 (30.15)	74.27 (32.18)	73.84 (29.80)	73.76 (33.46)	73.77 (30.17)	72.82 (28.87)	74.44 (34.51)	*

* means 100% identity.

### Characterization of prolyl endopeptidase features

**Figure 4:**
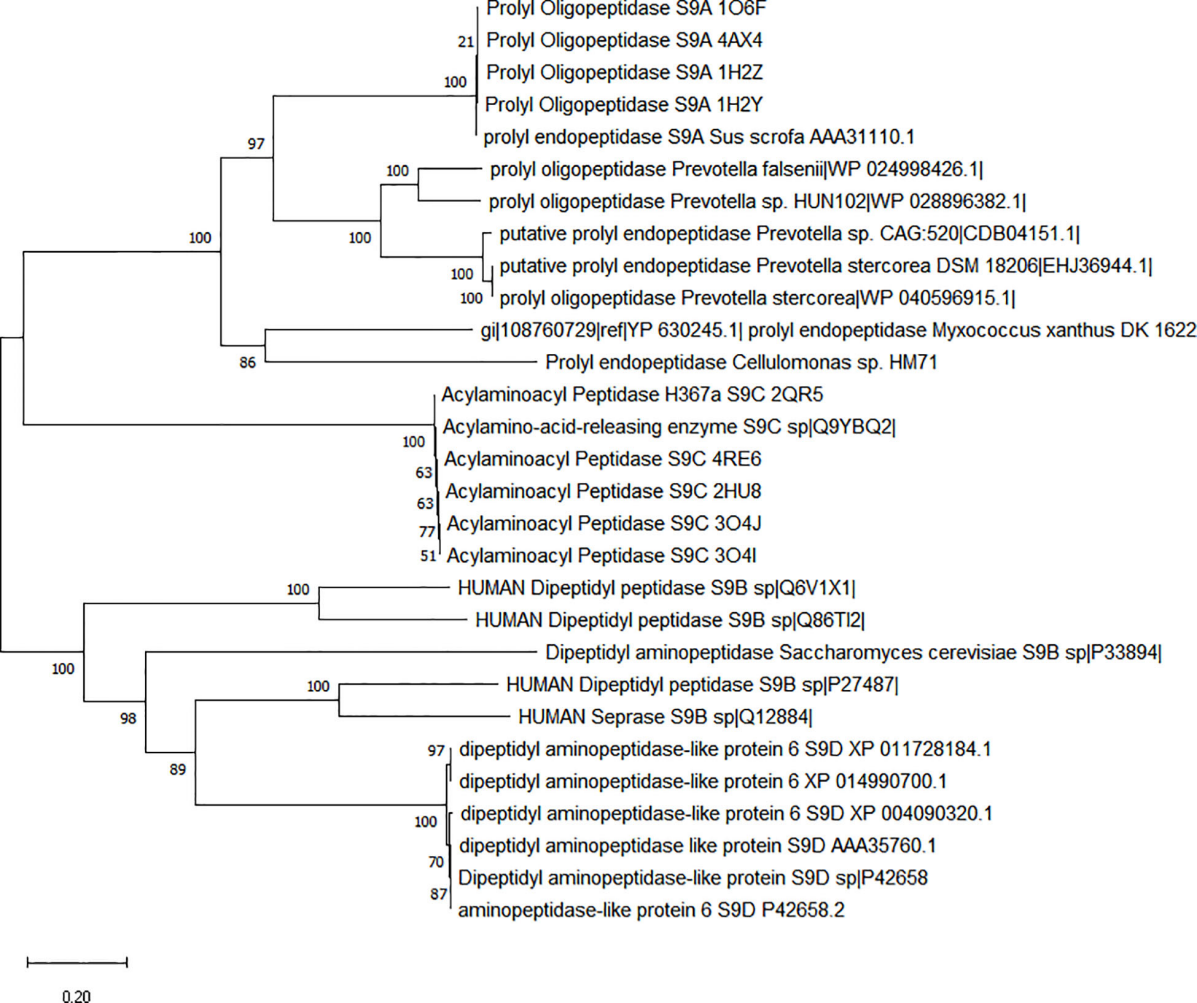
Phylogenetic characterization of Prolyl endopeptidase feature of *Cellulomonas* sp. HM71. Phylogenetic tree was constructed using the Neighbor-Joining (NJ) method with sequence of *Cellulomonas* sp. HM71 PEP and S9 Serine protease family members (S9A, S9B, S9C, and S9D). Numbers at the node represent bootstrap values. The bar at the bottom indicates the genetic evolutionary trajectory regarding genomic changes. The units of branch length are nucleotide substitutions per site.

Plate screening assays confirmed the gluten hydrolysis potential of *Cellulomonas* sp. HM71 (**Supplementary Figure S2**, **S3**). Time series degradation analysis of the antigenic gluten fraction (**Supplementary Figure S4**) also confirms previous claims (Kumar et al., 2018). Prolyl endopeptidase (PEP) activity was found to increase with microbial growth **(** **Supplementary Figure S5** **)**. All these results indicated the presence of gluten catabolic features within *Cellulomonas* sp. HM71 genome. Genome elucidation of *Cellulomonas* sp. HM71 identified two genetic features of sizes 2172bps and 2151bps, encoding Prolyl endopeptidase (EC 3.4.21.26) and putative prolyl oligopeptidase family protein, respectively. *Cellulomonas* sp. HM71 Prolyl endopeptidase shared an identity of 96.13% with prolyl oligopeptidase family serine peptidase of *Cellulomonas septica* in the non-redundant protein database, an identity of 37.79% (with 98% query coverage) was observed with well-characterized Prolylendopeptidases of *Myxococcus xanthus* in Protein databank protein database. CDD (CDD v3.19 -58235PSSMs) search identified the presence of a Prolyl oligopeptidase PreP conserved domain and also indicated it as a member of PreP superfamily. PFam database (Pfam_v33.1-18271 PSSMs) search indicated this protein as a member of the Peptidase_S9_N super family and alpha/beta hydrolases superfamily. Phylogenetic analysis of identified prolyl endopeptidase with the other members of the serine protease family (S9A, S9B, S9C, and S9D) indicates clustering of human gut prolyl endopeptidase sequences with the other members of the S9A serine family (**Figure 4**). Sequence alignment of the PEP proteins with characterized prolyl endopeptidase of *Aeromonas punctata* PEP (apPEP) and *Myoxococcus xanthus* PEP (mxPEP-zPP), indicates the presence of catalytically important residues (Trp582, Arg627, Asp 625, Ser541, and His660) numbering as per *Aeromonas punctata* PEP (Barik et al., 2021) (**Supplementary Figure S6**). The presence of conserved catalytic residues indicates that this protein could be involved in the gluten hydrolytic potential of *Cellulomonas* sp. HM71.

*Cellulomonas* sp. HM71 putative prolyl oligopeptidase family protein shared identity of 94% S9 family peptidase of *Cellulomonas septica* in the non-redundant protein database; while a very limited similarity of 36% (with only 33% sequence coverage) was observed with S9 peptidase from *Deinococcus radiodurans* R1.CDD (CDD v3.19 -58235PSSMs) search indicated the presence of a Dipeptidyl aminopeptidase/acylaminoacyl peptidase conserved domain and indicated it as a member of DAP2 superfamily. Pfam database (Pfam_v33.1-18271 PSSMs) search indicated this protein as a member of alpha/beta hydrolases superfamily. Phylogenetic analysis of identified prolyl endopeptidase with the other members of the serine protease family (S9A, S9B, S9C, and S9D) indicates clustering of human gut prolyl endopeptidase sequences with the other members of the S9B & S9D serine family (**Supplementary Figure S7**). As dipeptidyl aminopeptidase is involved in cleaving X-proline dipeptide from the N-terminus of protein, so it may have some role in antigenic gluten hydrolysis; however it needs to be studied in detail.

### Biosafety assessment of *Cellulomonas* sp. HM71

Bacterial toxicity analysis is essential to consider any bacterial strain as a potential probiotic. *Cellulomonas* sp. HM71 was tested for its toxicity and hemolytic activity. *Cellulomonas* sp. HM71 indicated no hemolytic activity in the blood agar plate assay after 24 hrs of incubation at 37°C (**Supplementary Figure S8**). Even cytotoxicity analysis demonstrated 83.22 ± 4.88% and 82.88 ± 3.91% Caco-2 cell viability after 24 hrs of exposure to the cell-free supernatant and cell lysate of *Cellulomonas* sp. HM71 respectively. Additionally, the gut isolate (cell suspension) showed 5.67% cellular adherence to Caco-2 cell after 24 hrs of exposure.

### Stress response physiology

**Figure 5:**
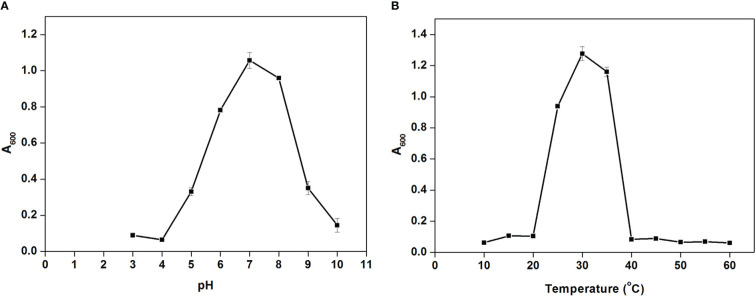
Growth pattern assessment of the *Cellulomonas* sp. HM71in Luria-Bertani broth with diverse pH (3-10 with an interval of 1.0 pH) **(A)** and temperature (10-60°C with an interval of 5°) **(B)** conditions for 24hrs with constant shaking at 200 rpm. Each point in the graph represents the mean of readings observed in triplicate experiments.

*Cellulomonas* sp. HM71 showed continued growth within the pH range of 5.0-8.0 (optimum growth pH = 8.0) (**Figure 5A**) and within the temperature range of 25°C-35°C (optimum growth temperature = 30°C) (**Figure 5B**). *Cellulomonas* sp. HM71 showed continued growth in the LB medium supplemented up to 3.0% NaCl (w/v) and 6.0% KCl (w/v). Similarly, *Cellulomonas* sp. HM71 showed growth in presence of various metals (Silver, Lead, Cadmium, and Potassium) and metalloid arsenic. *Cellulomonas* sp. HM71 also exhibited resistance to Cephalothin (30µg), Ceflnaxone (30µg), Ceftazidime (30µg), and Ofloxacin (2µg), moderate susceptibility to Amoxicillin (10µg), Bacitracin (10µg), and Lincomycin (2µg), and high susceptibility to Amikacin (10 and 30µg), Erythromycin (15µg), Novobiocin (30µg), Oxytetracycline (30µg), Vancomycin (30µg), Cefotaxime (30µg), and Netilin (30µg). Very limited growth suppression of 8.49 ± 1.65% was observed in the bile-enriched medium and was found to be negative for bile salt hydrolysis. *Cellulomonas* sp. HM71 did not show any significant growth suppression in the gastric conditions and intestinal conditions; however, a high concentration of lysozyme (100mg/L) suppressed the *Cellulomonas* sp. HM71 growth.

### Auto-aggregation and cell surface hydrophobicity

**Figure 6:**
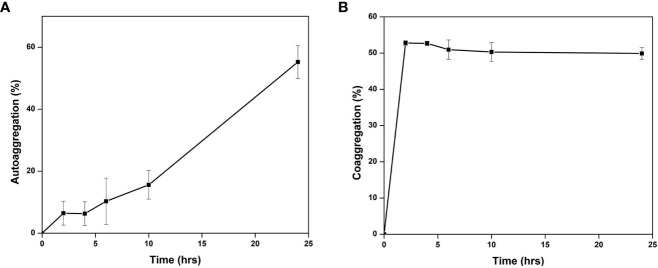
Auto-aggregation **(A)** and Co-aggregation **(B)** of *Cellulomonas* sp. HM71 with a pathogen mixture of *E.coli, Staphylococcus aureus*, and *Salmonella typhim*urium.

The gut isolate shows adherence to the epithelial cells and mucosa due to their auto-aggregation activity (Krausova et al., 2019). *Cellulomonas* sp. HM71 cells showed low adherence to toluene (22.41 ± 5.31%) which confirms the hydrophilic and electron-donating nature. *Cellulomonas* sp. HM71 showed 55.43 ± 1.71% auto-aggregation after 24 hrs, with auto-aggregation of 6.47 ± 4.85%, 6.28 ± 5.62%, 10.47 ± 4.61%, and 15.62 ± 4.85% after 2, 4, 6, and 10 hrs, respectively (**Figure 6A**). Auto-aggregation profile of the *Cellulomonas* sp. HM71 remained similar even after 24 hrs (**Supplementary Figure S9**); however, the hydrophobicity profile was found to increase after 24hrs (**Supplementary Figure S10**).

### Health-promoting properties of *Cellulomonas* sp. HM71

**Figure 7:**
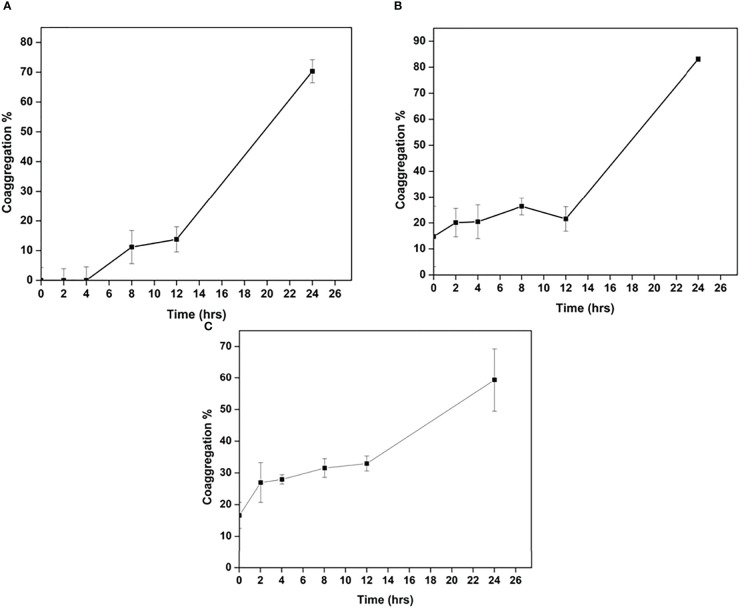
Co-aggregation of Cellulomonas sp. HM71 with Salmonella typhimurium **(A)**, Staphylococcus aureus **(B)**, and. E.coli **(C)**.

*Cellulomonas* sp. HM71 was found to co-aggregate with the pathogenic strains. The highest percentage of co-aggregation was 77.58 ± 1.36%, followed by 65.41 ± 6.79%, 64.86 ± 7.76%, 63.92 ± 7.69%, and 62.02 ± 3.57%, observed for pathogenic mixture of *Staphylococcus aureus* (MTCC No. 96)*, E.coli* (MTCC No. 443), *Salmonella typhi* (MTCC No. 98) and *Cellulomonas* sp. HM71 (**Figure 6B**). *Cellulomonas* sp. HM71 showed 70.33 ± 3.87%, 83.09 ± 0.93%, and 59.33 ± 9.89% coaggregation with *Salmonella typhi* (MTCC No. 98), *Staphylococcus aureus* (MTCC No. 96), and *E.coli* (MTCC No. 443), respectively (**Figure 7**). Disc diffusion assay showed an anti-pathogenic activity for *Cellulomonas* sp. HM71 against three pathogenic strains of *Staphylococcus aureus* (MTCC No. 96)*, E.coli* (MTCC No. 443), and *Salmonella typhi* (MTCC No. 98). A growth inhibition zone of 11.34 ± 2.31mm was observed for *Salmonella typhi* (MTCC No. 98), indicative of its protective nature against water borne infections. A growth inhibition zone of 8.00 ± 0.00mm and 10.67 ± 0.57mm was also observed for *E.coli* (MTCC No. 443) and *Staphylococcus aureus* (MTCC No. 96), respectively.

Anti-glycemic and anti-lipogenic effects are considered therapeutic targets to overcome diabetes Mellitus (Type-II), obesity, and pathological cardiovascular conditions (Salehi et al., 2020). Thus, α-amylase inhibition seems to be the prime therapeutic target. *Cellulomonas* sp. HM71 has shown 8.7 ± 2.28% inhibition in amylase activity. The isolated microbial culture shows 15.56 ± 0.36% inhibition of the lipase activity. Similarly, the *Cellulomonas* sp. HM71 showed cholesterol oxidizing activity. *Cellulomonas* sp. HM71 also showed a significant Lactase activity (0.62 units/mg), which could help overcome lactose indigestibility issues for lactose intolerant individuals. *Cellulomonas* sp. HM71 was found to possess 15.32 ± 0.07 units/mg bacterial pellet with 130.02 ± 0.02 units/mg alkaline phosphatase and acid phosphatase activity, respectively. Phosphatase activity could play an important role in cell proliferation and differentiation. This microbe was also found to have peroxidase activity (17.62 ± 0.02 units/mg) of the enzyme, which could help to overcome oxidative stress. Laccase is a multi-copper oxidase that was characterized to play a vital role in host health (Janusz et al., 2020). *Cellulomonas* sp. HM71 was also found to have laccase enzyme activity (0.0034 units/mg).

## Discussion

Human gut microbes play an important role in the maintenance of human health (Yadav and Chauhan, 2022). Microbial supplementation as therapeutics has advanced human health through disease management (Yadav and Chauhan, 2022). These probiotics as dietary supplements were found significant in fortifying human health (Wang et al., 2019). The major concern with the use of probiotics in any organism is its biosafety (Yadav and Chauhan, 2022). *Cellulomonas* sp. HM71 has been characterized for its potential to hydrolyze antigenic gluten peptides and could be employed as a probiotic for the treatment of gluten-derived celiac diseases (Kumar et al., 2018). However, it is yet to be characterized for its suitability as a probiotic strain. As various *Cellulomonas* sp*ecies* have already been assessed for their probiotic potential (http://www.indogulfgroup.com/animal-probiotics/Cellulomonas-cartae.php). However, information about the *Cellulomonas* strains as probiotics is still evolving (Hong et al., 2009). Hereby, in the present study, *Cellulomonas* sp. HM71 was characterized for its physiology, genomic architecture, and probiotic potential. Phenotypic and carbohydrate utilization profile of *Cellulomonas* sp. HM71 is in line with the substrate utilization characteristic of other *Cellulomonas* species. The whole-genome analysis of the human gut isolate indicated the presence of COGs associated with the general adaptive and metabolic mechanisms required for the microbial survival within the human body, thus, suggesting a strong affiliation to survive and thrive within the human host (Yadav et al., 2020; Yadav et al., 2021). The *Cellulomonas* sp. HM71 was found to possess no pathogenic islands indicating its safety considerations for probiotic features (Fu et al., 2022). The microbe contains a total of 30 CAZymes. The *Cellulomonas* strains have unique biocatalytic characteristics (Pourcher et al., 2001). Microbes have to face various stressful conditions within the human body such as pH, temperature, salt, and gastric conditions. Thus, a probiotic bacterium should possess significant features to resist all these stressful conditions. *Cellulomonas* strain survives well within the host’s body (Ndongo et al., 2018). Likewise, *Cellulomonas* sp. HM71 was identified to thrive in high salt, variable pH, and temperature conditions indicating its suitability to survive in a highly variable gut ecosystem. Bile salts are a threat to microbial growth (Urdaneta and Casadesús, 2017) which bacteria combats by expressing genes responsible for bile salt resistance (Ruiz et al., 2013).

Although slightly less growth was observed in bile salt, *Cellulomonas* sp. HM71 showed its bile tolerance property. The species-specific acid tolerance might strengthen the potential of *Cellulomonas* sp. HM71 to survive in acidic conditions (Maki et al., 2009) similar to the microbes well-adapted to acidic environments (Guan and Liu, 2020). The *Cellulomonas* sp. HM71 was found to adapt within the host’s gut. Thus, its survivability under diverse conditions makes it suitable to be used for human consumption. The cytotoxicity analysis of *Cellulomonas* sp. HM71 against Caco-2 cells indicates its safety and non-toxicity. Even it did not show any hemolytic activity. An ideal probiotic microbe must possess the tendency of auto-aggregation and co-aggregation which makes it suitable as an anti-pathogenic agent (Collado et al., 2008). Probiotics due to their auto-aggregation property form a protective defense layer to inhibit the entry of pathogens (Monteagudo-Mera et al., 2019) while co-aggregation tendency enables them to bind to the pathogens and eradicate them directly. The differential auto-aggregation and co-aggregation percentages are due to diverse internal and environmental factors of *Cellulomonas* sp. HM71 (Stevens et al., 2015). Thus, this microbe may be efficient to protect the host against incoming pathogens. Probiotics can overcome the drawbacks of traditional therapeutics by combating their antibiotic-resistant profile and avoiding the spread of antibiotic resistance to other species of the microbe (Markowiak and Śliżewska, 2017).

The bacterial isolate can eradicate the pathogens by reducing their survival within the human host. The gut isolate was found to suppress the growth of the pathogenic strain *Salmonella typhi* indicating the role of microbe in protecting the host against pathogens. Probiotics must adhere to the host epithelial cells to form a defense layer against the incoming pathogens due to their auto-aggregation capability (Monteagudo-Mera et al., 2019). The adherence capacity of a bacterial cell is dependent on its nature. The low adherence capacity of the isolated bacterium to toluene (22.41 ± 5.31%) confirms its hydrophilic as well as electron-donating behavior. The gut isolate was found to show various enzymatic activities such as anti-lipase, anti-amylase, cholesterol-removing ability, laccase, lactase, and protease that help the microbe in providing health benefits to the host. The polyphenols in the diet induce the hyperglycemic effect as they inhibit the digestive enzymes by binding to the glucose transporters. The glucose formed through dietary metabolism causes a rise in blood glucose due to the action of the digestive enzyme α-amylase. Thus, α-amylase needs to be inhibited during Type-II diabetes. Phosphatases are enzymes that are significant for cell proliferation and differentiation. Both alkaline and acid phosphatase activities were observed within the gut isolate. The significant activity of the peroxidase enzyme helps bacteria to resist oxidative stress. Lactose indigestion may lead to lactose intolerance, thus, lactase is necessary to form glucose from lactose for the treatment of lactose intolerance. *Cellulomonas* sp. HM71 possesses significant lactase activity making it suitable for possibly treating lactose indigestion. Dietary polyphenols also form a major part of the diet. The presence of laccase activity confirms the role of *Cellulomonas* sp. HM71 in polyphenol metabolism. Obesity is a major threat to the human body due to excessive fat deposition (Bryson et al., 2009). The anti-lipase activity is significant to reduce fat absorption. The presence of good lipase activity in *Cellulomonas* sp. HM71 makes it significant to treat obesity through reduced fat absorption. *Cellulomonas* sp. HM71 possesses the ability to remove cholesterol and thus can be used to treat hypercholesterolemia. Conclusively, *in vitro* experimentation confirmed the safety, efficacy, and metabolic efficiency of *Cellulomonas* sp. HM71 and indicates it as a candidate probiotic strain. Supported by data, a high efficacy of the gut isolate *Cellulomonas* sp. HM71 in metabolizing gluten peptides as well as its probiotic potential strengthens its potential use as a candidate strain in relieving the symptoms of celiac disease. However,before its commercial deployment, its probiotic efficacy must be validated in animal models as well as through clinical trials for celiac disease.

## Data availability statement

The sequencing data is publicly available under NCBI Bio project ID: PRJNA895182 and Biosample ID: SAMN31495590.

## Ethics statement

The studies involving human participants were reviewed and approved by Institutional Human Ethical Committee, Maharshi Dayanand University, Rohtak. The patients/participants provided their written informed consent to participate in this study.

## Author contributions

NC designed the study and experiments. NC and MY wrote the manuscript. MY and TK carried out the experiments. RP and RM performed genome sequencing. NC, MY, and RM analyzed the data. All authors edited the manuscript and approved the final draft of the manuscript.

## Supplementary material

Supplementary Method SM1 Genome assembly of the Cellulomonas sp. HM71 (Yadav et al., 2022).

Supplementary Method SM2 Prolyl endopeptidase activity and gluten hydrolytic potential of Cellulomonas sp. HM71 (Kumar et al., 2018).

Supplementary Method SM3 Bio-Safety evaluation of the Cellulomonas sp. HM71 (Yadav et al., 2022).
