# Supplementary material for: Characterization of Cellulomonas sp. HM71 as potential probiotic strain for human health
Source: Front Cell Infect Microbiol. 2023 Jan 13;12:1082674. doi: 10.3389/fcimb.2022.1082674 (PMC9880229; doi:10.3389/fcimb.2022.1082674)
Supplement: Supplementary Method SM1 — Genome assembly of the Cellulomonas sp. HM71 (Yadav et al., 2022). [file DataSheet_1.pdf]

Characterization of *Cellulomonas* sp. HM71 as potential probiotic strain for human health

Monika Yadav[1], Tarun Kumar[1], Ranjeet Maurya[2,3], Rajesh Pandey[2†], and Nar Singh Chauhan[1*]

[1]Department of Biochemistry, Maharshi Dayanand University, Rohtak, Haryana, India

[2]Integrative GENomics of Host-PathogEn (INGEN-HOPE) laboratory, CSIR-Institute of Genomics and Integrative Biology (CSIR-IGIB), Mall Road, Delhi-110007, India

[3]Academy of Scientific and Innovative Research (AcSIR), Ghaziabad-201002, India

*Corresponding author

Nar Singh Chauhan (nschauhan@mdurohtak.ac.in)

Running Title: Probiotic potential of *Cellulomonas* sp. HM71

Number of Words: 7003

Number of Figures: 7

Number of Tables: 3

A. Supplementary Methods

Supplementary Method SM1: Genome assembly of the *Cellulomonas* sp. HM71 (Yadav et al., 2022).

Raw reads were quality checked using FASTQC v0.11.9 (http://www.bioinformatics.babraham.ac.uk/projects/fastqc) and fastQValidator v0.1.1 (https://github.com/statgen/fastQValidator). Removal of contaminated reads was performed to get the error corrected reads. The SPAdes v3.15.1 assembler was used for the de-novo assembly which uses an automatic k-mer optimization approach and is thereby a good tool for bacterial genome assembly. It uses Bayes Hammer to perform read error correction on each data set and MismatchCorrector - a post-processing tool, to reduce the number of mismatches in assembly using the BWA tool. Further, the BUSCO v5.0.0 assessment tool was used with the latest bacterial orthologous catalog (bacteria_odb10) for analyzing the completeness of a set of predicted genes in bacterial genome assemblies (https://busco.ezlab.org/).

Supplementary Method SM2: Prolyl endopeptidase activity and gluten hydrolytic potential of *Cellulomonas* sp. HM71 (Kumar et al., 2018).

Plate screening of gluten hydrolytic activity was performed by applying the culture on LB agar medium plates supplemented with 1% gluten (w/v). The bacterial suspension (100µl of 1.0OD_{600nm} culture was also applied to the screening medium (Luria–Bertani broth (LB) supplemented with 1% gluten) using well-diffusion assay. Screening medium plate was incubated at 37°C and observed for the presence of a hydrolytic halo zone surrounding the colony.The assay was carried out in triplicate and sterile PBS was used as control (Kumar et al., 2018). Antigenic fraction of gluten was prepared from wheat gluten (Kumar et al., 2018). Gluten catabolic potential of *Cellulomonas* sp. HM71 was checked against both synthetic substrate and gluten antigenic fraction. Catabolism of antigenic gluten fraction was observed in a reaction mixture (20 ml) containing actively growing culture(A_{600nm}=4.0), antigenic peptide (5 mg/ml) in PBS (pH 7.4). Reaction mixture was incubated at 37°C at constant shaking and fractions were collected at different time intervals (0, 0.5, 1, 2, 4, 8, 12, 16, and 24 h). Fractions were used to detect the degradation of antigenic fraction using the ninhydrin reagent. A few drops of ninhydrin reagent were added to 1ml of the antigenic fraction and incubated at 80-100°C for 4-7 mins. The blue color so formed was read at 570nm. To confirm the role of human gut microbe in gluten catabolism, the fraction collected at different time intervals (0, 0.5, 1, 2, 4, 8, 12, 16, and 24 h) was centrifuged at 13,000 rpm for 5 mins and the microbial pellet was used for checking biocatalytic activity against the Z-Gly-Pro-pNA substrate. The bacterial pellet was suspended in PBS and 20mM Z-Gly-Pro-pNA (Sigma Aldrich) substrate was used. The reaction mixture was incubated at 37°C and absorbance was taken at 405nm (Kumar et al., 2018).

Supplementary Method SM3: Bio-Safety evaluation of the *Cellulomonas* sp. HM71 (Yadav et al., 2022).

(a) Adherence assay: Caco-2 cells were cultured in Dulbecco's modified Eagle's medium (DMEM, Himedia) supplemented with 10% fetal bovine serum (Gibco, Brazil origin), 100 U/ml penicillin,100 µg/ml streptomycin and 0.25 µg/ml amphotericin B (Gibco) at 37˚C in 5% CO_2. Caco-2 cells were seeded in a 24 well cell culture plate (Nunc) at 1.0×10^5 cells/cm^2 and cultured for 2 days. Before the adherence assay, Caco-2 cell monolayers were washed three times with PBS (Gibco), and the medium was replaced with antibiotic-free DMEM. Each well in cell culture plate was inoculated with 100 µl of 1.0 $OD600_{nm}$ culture and incubated for 2 h at 37˚C in 5% CO_2. After 2 h of incubation, DMEM medium containing non-adhered bacterial cells was collected from each well and the monolayers were washed three times with PBS, and 100 µl of 1% Triton-X 100 (Sigma-Aldrich) was added to obtained homogenate. 100µl of 10^{-4} dilutions of homogenate and bacteria suspended medium were plated on PB agar plates. Colony forming units (CFUs) were counted and total number of CFUs incubated with cell monolayers were thus obtained by the addition of both the counts. The percentage of bacteria that adhered to the plate was then calculated. All experiments were performed three times independently.

(b) Cytotoxicity Assay: Caco-2 cells were seeded in a 96-well plate (3×10^4 cells/well) and incubated at 37 °C with 5% CO2 for 24 h. Bacterial culture were grown for 18 hrs at 37 °C and centrifuged at 13000 rpm for 5 minutes. Microbial pellet was sonicated at 55% amplitude for 5 minutes (10s on and 5s off cycle and centrifuged at 13000 rpm for 5 minutes at 4°C. Supernatant was then collected and filter sterilized with 0.2µm syringe filters and kept at 4°C until used. The lysate was filter sterilized with 0.2µm syringe filters and kept at 4°C until used. Medium was removed from cell culture plate and 50µl of DMEM was added to each well prior to the experiments. A 50 µl of sterilized cell free supernatant of bacterial culture was added to each well and incubated at 37 °C for 24 h. To analyze the effect of bacterial lysate, A 50 µl of sterilized bacterial lysate was added separately to each well and incubated at 37 °C for 24 h. The mixtures were then removed, and 100µl of 0.5 mg/ml of 3-(4,5-dimethylthiasol-2-yl)-2,5-diphenyltetrazolium bromide (MTT) solution was added to each well followed by incubation for 3 h in darkness. The MTT solution was gently removed and a 100µl dimethyl sulfoxide (DMSO) was added to solubilize formazan crystals. The absorbance was measured at $\lambda = 570$ nm. DMEM mixed with LB was used as control. Experiments were conducted in triplicate. The Caco-2 cell viability was calculated using the following equation: % Cell viability = [(ODsample × 100)/ODcontrol].

B. Supplementary Figure

Supplementary Figure S1. COG categories observed within the genome of *Cellulomonas* sp. HM71 where R codes for General function prediction only, E codes for Amino acid transport and metabolism, C codes for Energy production and conversion, G codes for Carbohydrate transport and metabolism, J codes for Translation, ribosomal structure and biogenesis, P codes for Inorganic ion transport and metabolism, L codes for Replication, recombination and repair, M codes for Cell wall/membrane/envelope biogenesis, H codes for Coenzyme transport and metabolism, F codes for Nucleotide transport and metabolism, K codes for Transcription, T codes for Signal transduction mechanisms, I codes for Lipid transport and metabolism, O codes for Posttranslational modification, protein turnover, chaperones, V codes for Defense mechanisms, Q codes for Secondary metabolites biosynthesis, transport and catabolism, U codes for Intracellular trafficking, secretion, and vesicular transport, D codes for Cell cycle control, cell division, chromosome partitioning, S codes for Function unknown, N codes for Cell motility, and Z codes for Cytoskeleton.

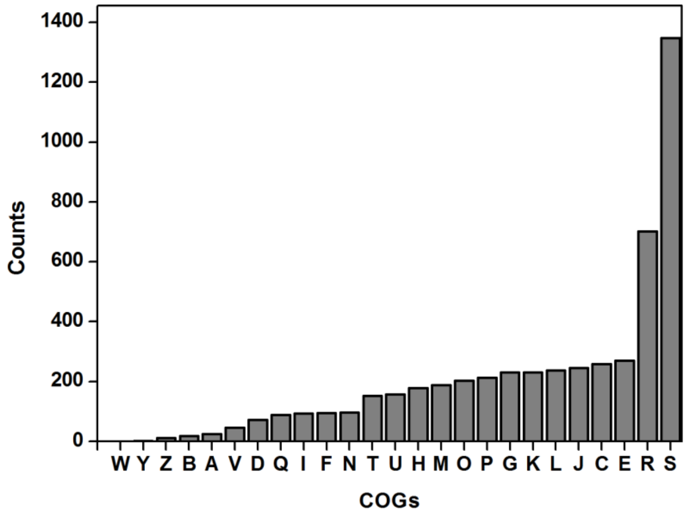

Supplementary Figure S2. Agar plate assay showing the gluten hydrolytic activity of *Cellulomonas* sp. HM71 in screening medium (Luria–Bertani broth (LB) supplemented with 1% gluten) after the incubation of 24hrs at 37ºC.

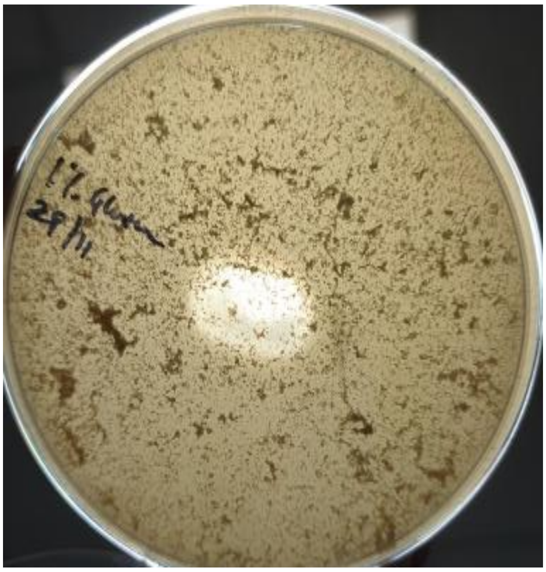

Supplementary Figure S3. Well Diffusion assay showing the gluten hydrolytic activity of *Cellulomonas* sp. HM71 in screening medium (Luria–Bertani broth (LB) supplemented with 1% gluten) after the incubation of 24hrs at 37°C. The assays were carried out in triplicate, and sterile PBS was used in the control well.

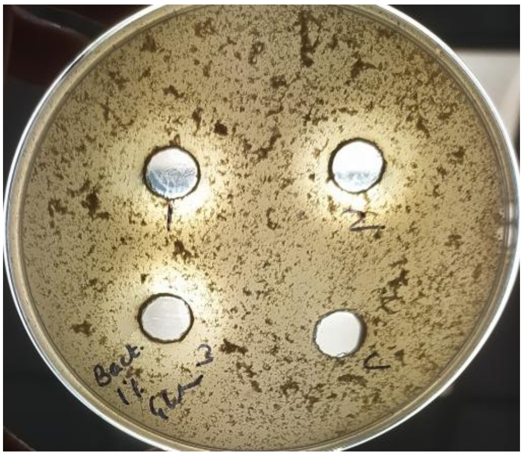

Supplementary Figure S4. Degradation of the gluten antigenic fraction by *Cellulomonas* sp. HM71. The microbe was grown in an assay medium (Luria–Bertani broth (LB) supplemented with 1% gluten antigenic fraction). Fractions were collected at different time intervals for the quantitative estimation of gluten degradation. Each point in the graph is the mean of three different replicates.

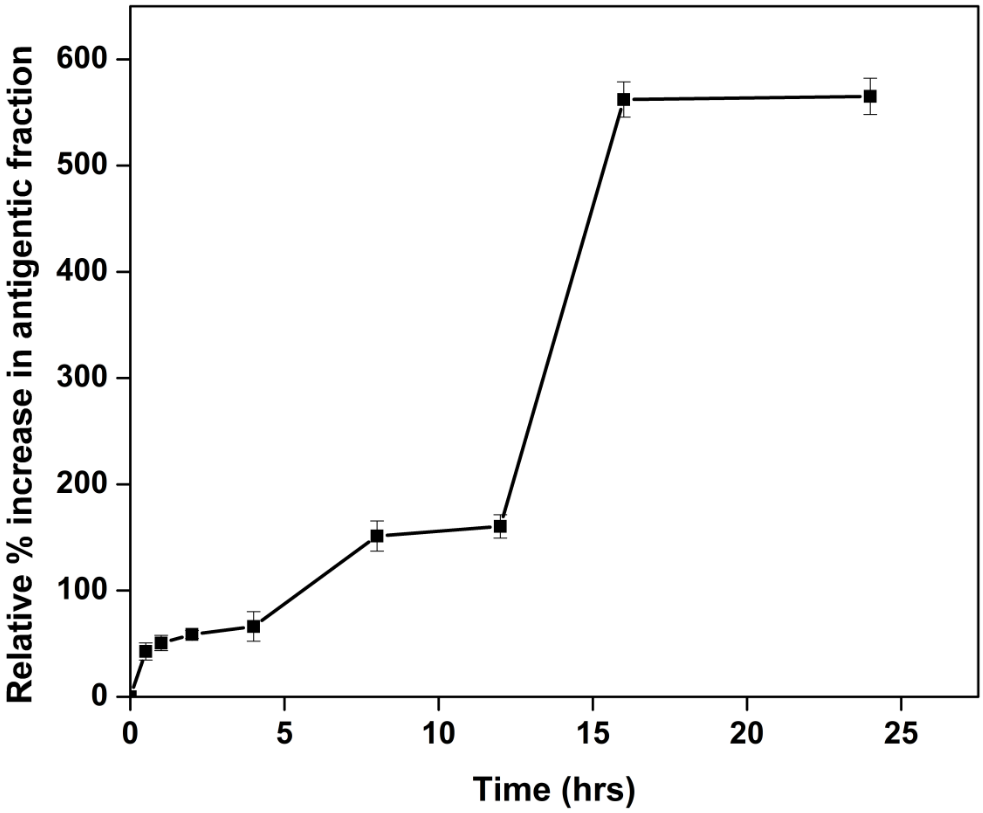

Supplementary Figure S5. Prolyl endopeptidase activity of *Cellulomonas* sp. HM71 at different time intervals. The microbe was grown in Luria–Bertani broth (LB). Microbial fractions were taken at different time intervals (0, 0.5, 1, 2, 4, 8, 12, 16, and 24 h) for the quantitative estimation of PEP activity. Growth pattern (a) and PEP activity of the human gut isolate *Cellulomonas* sp. HM71 was observed at different time intervals (b). Each point in the graph is the mean of three different replicates.

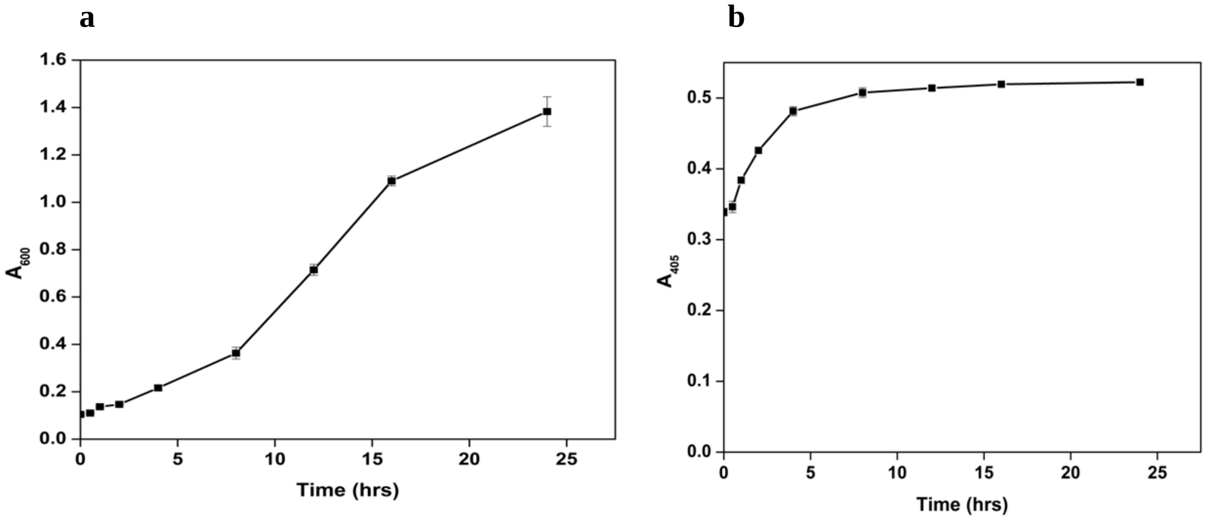

Supplementary Figure S6. Sequence alignment of *Cellulomonas* sp. HM71 prolyl endopeptidase with their well-characterized homologous of *Aeromonas punctate (apPEP)* and *Myoxococcus xanthus (mxPEP)*

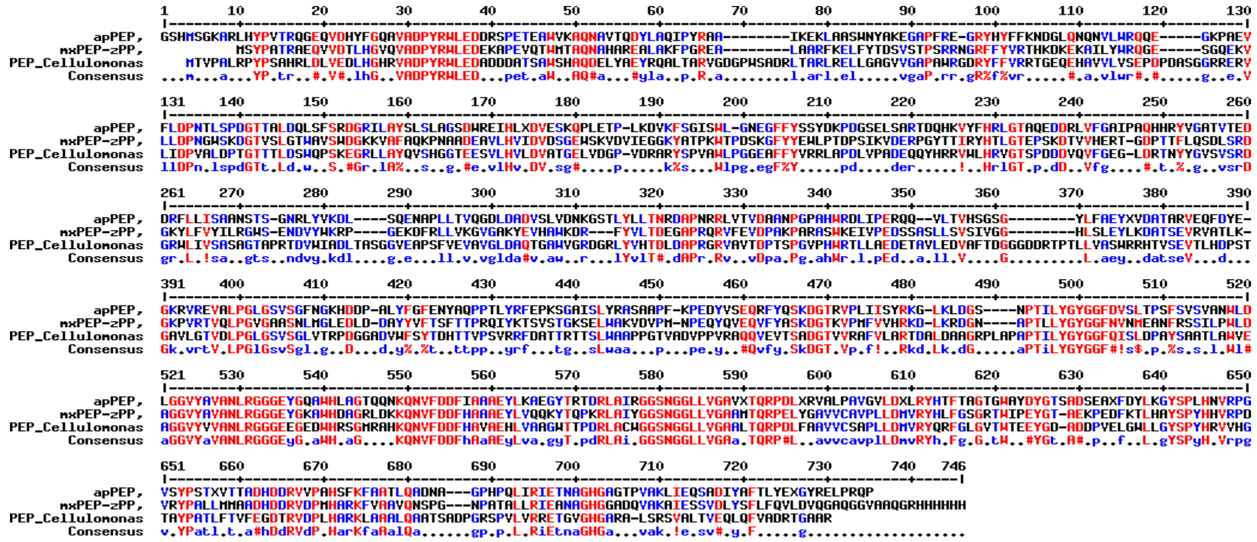

Supplementary Figure S7. Phylogenetic characterization of prolyl oligopeptidase feature of *Cellulomonas* sp. HM71. Phylogenetic tree was constructed with the neighbor-joining method using the prolyl oligopeptidase sequence of *Cellulomonas* sp. HM71 and Serine protease family members using MEGAX software. Numbers at the node represent bootstrap values. The bar at the bottom indicates the genetic evolutionary trajectory regarding genomic changes. The units of branch length are nucleotide substitutions per site.

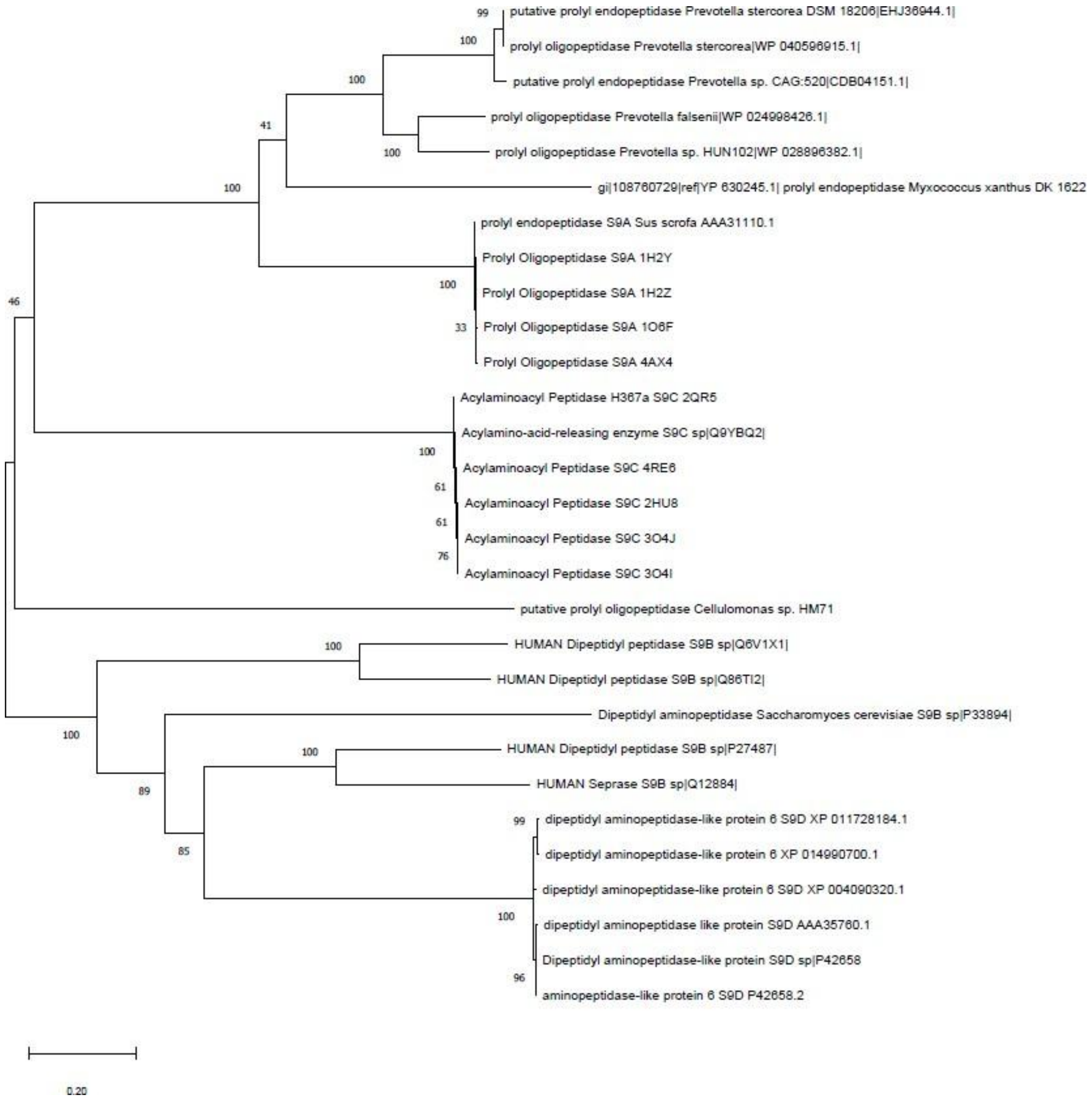

Supplementary Figure S8. The blood agar plate (5% v/v) shows the hemolytic activity of *Cellulomonas* sp. HM71 after 24 hrs of incubation at 37°C

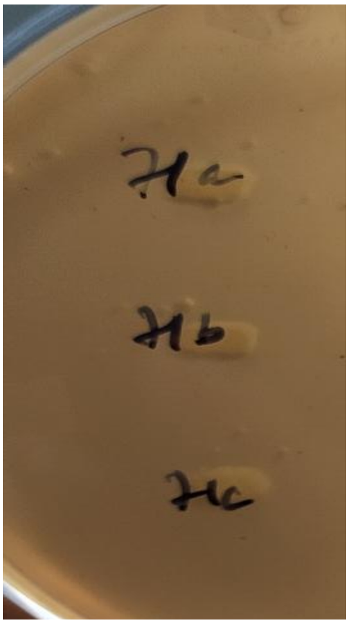

Supplementary Figure S9. Autoaggregation of *Cellulomonas* sp. HM71 at different time intervals within 36 hrs of incubation. Each point in the graph is the mean of three different replicates.

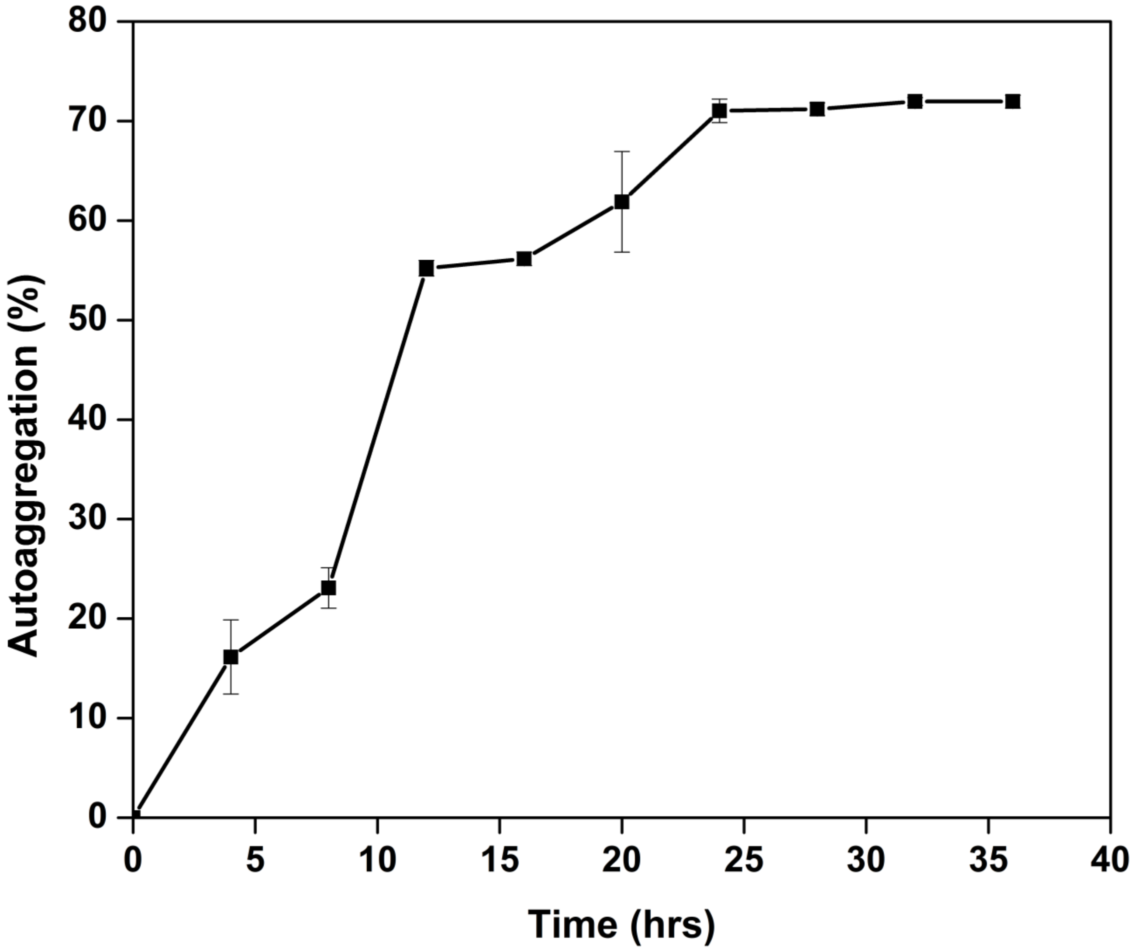

Supplementary Figure S10. Cell surface hydrophobicity of *Cellulomonas* sp. HM71 at different time intervals within 36 hrs of incubation. Each point in the graph is the mean of three different replicates.

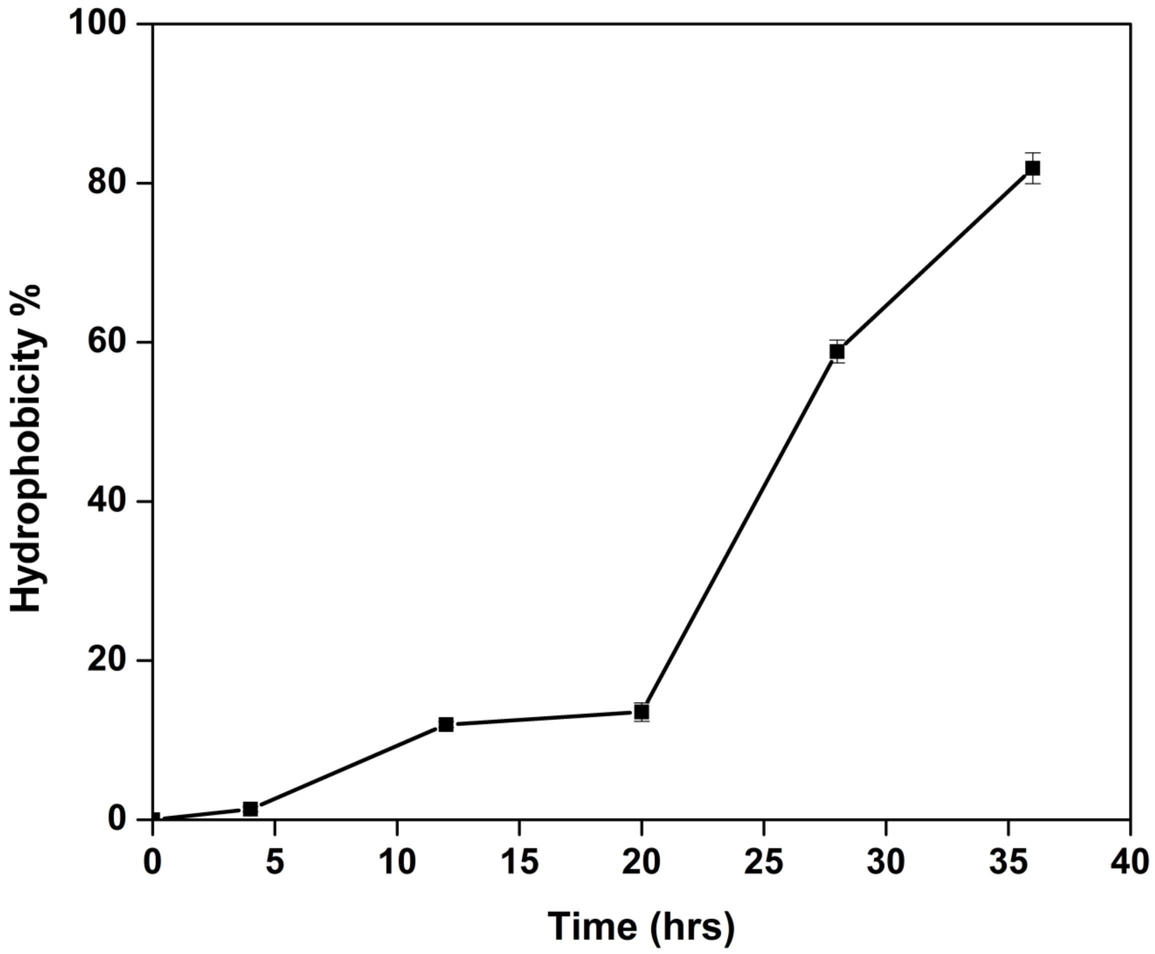

C. Supplementary Tables

Supplementary Table S1. Genomic features of *Cellulomonas* sp. HM71 and other *Cellulomonas* strains used for comparative genome analysis

Organism	Strain	Isolation source	Genome Size (Mb)	CDS	tRNA	rRNA	Genome Reference (NCBI ID)
Cellulomonas fimi	ATCC 484	Soil	4.27	3758	46	6	CP002666.1
Cellulomonas fimi	strain NCTC7547	Soil	4.27	3758	46	6	LR134387.1
Cellulomonas iranensis	strain ZJW-6	Soil	4.05	3532	45	6	CP084585.1
Cellulomonas flavigena	DSM 20109	Soil	4.12	3649	45	6	CP001964.1
Cellulomonas gilvus	ATCC 13127	Bovine feces	3.53	3147	45	6	CP002665.1
Cellulomonas shaoxiangyii	strain Z28	Feces of the Tibetan antelopes	3.91	3392	45	9	CP039291.1
Cellulomonas taurus	strain P40-2	Livestock	3.44	3105	46	9	CP051884.1
Oerskovia	sp. KBS0722	Soil	4.36	3800	53	12	CP042174.1
Isoptericola dokdonensis	DS-3	Soil	3.83	3392	50	12	CP014209.1

Supplementary Table S2. Substrate utilization profile of *Cellulomonas* sp. HM71 in comparison to phylogenetically related *Cellulomonas* strains

Sr.No.	Substrate	*Cellulomonas sp.* HM71	*Cellulomonas chitinilytica* XBU-b	*Cellulomonas fimi* 133	*Cellulomonas flavigena* 134	*Cellulomonas algicola* TKZ-21	*Cellulomonas biazotea* 127	*Cellulomonas cellasea* 124	*Cellulomonas uda* 136
1	Lactose	+	ND	ND	ND	ND	+	ND	ND
2	Xylose	+	+	ND	ND	ND	ND	ND	+
3	Maltose	+	ND	ND	ND	ND	ND	ND	ND
4	Fructose	+	+	ND	ND	+	ND	ND	+
5	Dextrose	+	+	+	ND	ND	+	+	+
6	Galactose	+	ND	ND	ND	+	ND	ND	+
7	Raffinose	ND	ND	ND	ND	ND	ND	ND	ND
8	Trehalose	+	ND	ND	ND	ND	ND	ND	ND
9	Melibiose	ND	ND	ND	ND	ND	ND	ND	ND
10	Sucrose	+	ND	ND	ND	ND	ND	ND	+
11	L-arabinose	+	ND	ND	ND	ND	ND	+	+
12	Mannose	+	ND	ND	ND	ND	ND	ND	+
13	Inulin	+	ND	ND	ND	ND	ND	ND	ND
14	Sodium glucan	ND	ND	ND	ND	ND	ND	ND	ND
15	Glycerol	+	ND	ND	ND	ND	ND	ND	ND
16	Salicin	+	ND	ND	ND	ND	ND	ND	ND
17	Dulcitol	ND	ND	ND	ND	ND	ND	ND	ND
18	Inositol	ND	ND	ND	ND	ND	ND	ND	ND
19	Sorbitol	ND	ND	ND	ND	ND	ND	ND	ND
20	Mannitol	ND	ND	+	+	ND	ND	+	+
21	Adonitol	ND	ND	ND	ND	ND	ND	ND	ND
22	Arabitol	ND	ND	ND	ND	ND	ND	ND	ND
23	Erythritol	ND	ND	ND	ND	ND	ND	ND	ND
24	α-Methyl-D	ND	ND	ND	ND	ND	ND	ND	ND
25	Rhamnose	+	ND	ND	ND	ND	ND	ND	ND
26	Cellobiose	+	+	ND	ND	ND	ND	ND	+
27	Melezitose	+	ND	ND	ND	ND	ND	ND	ND
28	α-Methyl-	ND	ND	ND	ND	ND	ND	ND	ND

	D-mannoside								
29	Xylitol	ND	ND	ND	ND	ND	ND	ND	ND
30	ONPG	ND	ND	ND	ND	ND	ND	ND	ND
31	Esculin hydrolysis	+	+	ND	+	ND	ND	ND	+
32	D-arabinose	+	ND	ND	ND	ND	ND	+	ND
33	Citrate utilization	ND	ND	ND	ND	ND	ND	ND	ND
34	Malonate utilization	ND	ND	ND	ND	ND	ND	ND	ND
35	Sorbose	ND	ND	ND	ND	ND	ND	ND	ND

***ND here refers to 'Not Detected'.**

Supplementary Table S3. BUSCO assessment of *Cellulomonas* sp. HM71

**Assembly**	***Cellulomonas* sp. HM71 contigs**
# contigs (>= 0 bp)	942
# contigs (>= 1000 bp)	144
# contigs (>= 5000 bp)	96
# contigs (>= 10000 bp)	77
# contigs (>= 25000 bp)	50
# contigs (>= 50000 bp)	20
Total length (>= 0 bp)	3796812
Total length (>= 1000 bp)	3579967
Total length (>= 5000 bp)	3485008
Total length (>= 10000 bp)	3362657
Total length (>= 25000 bp)	2920945
Total length (>= 50000 bp)	1836670
# contigs	239
Largest contig	185041
Total length	3643765
GC (%)	62.14
N50	51791
N75	29034
L50	20
L75	44
# N's per 100 kbp	4.94

Supplementary Table S4. Subsystem features related to antibiotic resistance, oxidative stress, and heat tolerance within *Cellulomonas* sp. HM71 genome

Level 1	Level 2	Level 3	Functions	Hits
Virulence, Disease and Defense	Resistance to antibiotics and toxic compounds	Cobalt-zinc-cadmium_resistance	Cation efflux system protein CusC precursor	1
Virulence, Disease and Defense	Resistance to antibiotics and toxic compounds	Multidrug Resistance Efflux Pumps	Acriflavin resistance protein	2
Virulence, Disease and Defense	Resistance to antibiotics and toxic compounds	Arsenic resistance	Arsenate reductase (EC 1.20.4.1)	8
Virulence, Disease and Defense	Resistance to antibiotics and toxic compounds	Arsenic resistance	Arsenical resistance operon repressor	1
Virulence, Disease and Defense	Resistance to antibiotics and toxic compounds	Arsenic resistance	Arsenical-resistance protein ACR3	1
Virulence, Disease and Defense	Resistance to antibiotics and toxic compounds	Beta-lactamase	Beta-lactamase	1
Virulence, Disease and Defense	Resistance to antibiotics and toxic compounds	Beta-lactamase	Beta-lactamase class C and other penicillin binding proteins	1
Virulence, Disease and Defense	Resistance to antibiotics and toxic compounds	Cobalt-zinc-cadmium resistance	Cobalt-zinc-cadmium resistance protein	1
Virulence, Disease and Defense	Resistance to antibiotics and toxic compounds	Cobalt-zinc-cadmium resistance	Cobalt-zinc-cadmium resistance protein CzcD	2
Virulence, Disease and Defense	Resistance to antibiotics and toxic compounds	Copper homeostasis	Copper-translocating P-type ATPase (EC 3.6.3.4)	6
Virulence, Disease and Defense	Resistance to antibiotics and toxic compounds	Copper homeostasis: copper tolerance	Cytoplasmic copper homeostasis protein cutC	2
Virulence, Disease and Defense	Resistance to antibiotics and toxic compounds	Cobalt-zinc-cadmium resistance	Heavy metal RND efflux outer membrane protein, CzcC family	1
Virulence, Disease and Defense	Resistance to antibiotics and toxic compounds	Copper homeostasis	Multicopper oxidase	3
Virulence, Disease and Defense	Resistance to antibiotics and toxic compounds	BlaR1 Family Regulatory Sensor-[illegible]cer	Peptidase M48, Ste24p precursor	1

		Disambiguation		
Virulence, Disease and Defense	Resistance to antibiotics and toxic compounds	Cobalt-zinc-cadmium resistance	Transcriptional regulator, MerR family	1
Virulence, Disease and Defense	Resistance to antibiotics and toxic compounds	Methicillin resistance in *Staphylococci*	Undecaprenyl-phosphate N-acetylglucosaminyl 1-phosphate transferase (EC 2.7.8.-)	1
Virulence, Disease and Defense	Resistance to antibiotics and toxic compounds	Resistance to Vancomycin	Vancomycin B-type resistance protein VanW	1
Stress Response	Oxidative stress	Protection from Reactive Oxygen Species	Catalase (EC 1.11.1.6)	2
Stress Response	Oxidative stress	Glutathione: Biosynthesis and gamma-glutamyl cycle	Gamma-glutamyltranspeptidase (EC 2.3.2.2)	2
Stress Response	Oxidative stress	Glutathione: Redox cycle	Glutaredoxin	1
Stress Response	Oxidative stress	Oxidative stress	Iron-binding ferritin-like antioxidant protein	1
Stress Response	Oxidative stress	Glutathione analogs: mycothiol	L-cysteine:1D-myo-inositol 2-amino-2-deoxy-alpha-D-glucopyranoside ligase MshC	1
Stress Response	Oxidative stress	Glutathione analogs: mycothiol	Mycothiol S-conjugate amidase Mca	2
Stress Response	Oxidative stress	NADPH:quinine oxidoreductase 2	NADPH:quinone oxidoreductase 2	1
Stress Response	Oxidative stress	Redox-dependent regulation of nucleus processes	Nicotinate phosphoribosyltransferase (EC 2.4.2.11)	2
Stress Response	Oxidative stress	Regulation of Oxidative Stress Response	RNA polymerase sigma factor	11

Stress Response	Oxidative stress	NADPH:quinine oxidoreductase 2	Redox-sensing transcriptional regulator QorR	1
Stress Response	Oxidative stress	Oxidative stress	Redox-sensitive transcriptional regulator (AT-rich DNA-binding protein)	2
Stress Response	Oxidative stress	Regulation of Oxidative Stress Response	Superoxide dismutase [Mn] (EC 1.15.1.1)	3
Stress Response	Oxidative stress	Oxidative stress	Zinc uptake regulation protein ZUR	1
Stress Response	Heat shock	Heat shock dnaK gene cluster extended	Chaperone protein DnaJ	2
Stress Response	Heat shock	Heat shock dnaK gene cluster extended	Ribonuclease PH (EC 2.7.7.56)	2
Stress Response	Heat shock	Heat shock dnaK gene cluster extended	Translation elongation factor LepA	1
Stress Response	Heat shock	Heat shock dnaK gene cluster extended	tmRNA-binding protein SmpB	1

Supplementary Table S5. Genes related to antibiotic and metal resistance within *Cellulomonas* sp. HM71 genome as identified through RAST-server

**Gene function**	**Pathway involved**	**Hits**
Glyoxalase/bleomycin resistance protein/dioxygenase		10
putative branched-chain amino acid permease (azaleucine resistance protein AzlC)		1
Transcriptional regulator, MerR family	Cobalt-zinc-cadmium resistanc	4
UDP-glucose 6-dehydrogenase (EC 1.1.1.22)	Polymyxin resistance	2
DNA gyrase subunit B (EC 5.99.1.3)	Resistance to fluoroquinolones	1
DNA gyrase subunit A (EC 5.99.1.3)	Resistance to fluoroquinolones	1
Outer membrane component of tripartite multidrug resistance system		1
RND multidrug efflux transporter; Acriflavin resistance protein	Multidrug Resistance Efflux Pu	1
small multidrug resistance family (SMR) protein		1
Copper resistance protein CopD / Cytochrome c oxidase caa3-type assembly factor (unrelated to Cox11-CtaG family)	Copper homeostasis	2
Tellurium resistance protein TerD		3
toxic anion resistance family protein		1
Cobalt/zinc/cadmium resistance protein CzcD		2
Organic hydroperoxide resistance protein	Oxidative stress	1
EmrB/QacA subfamily drug resistance transporter		1
Arsenical resistance operon repressor / Arsenate reductase (EC 1.20.4.4) thioredoxin-coupled, LMWP family		3
Translation elongation factor G	Tetracycline resistance	1
Chromate transport protein ChrA	Resistance to chromium compo	1
Multidrug resistance transporter, Bcr/CflA family	Copper homeostasis	1

Supplementary Table S6. List of CAZzymes determined within *Cellulomonas* sp. HM71 genome.

Sr. No.	Enzyme family	Activities in Family	Hits
1	Carbohydrate-Binding Module Family 32	Binding to galactose and lactose has been demonstrated for the module of *Micromonospora viridifaciens* sialidase. Binding to polygalacturonic acid has been shown for a *Yersinia* member. Binding to LacNAc (β-D-galactosyl-1,4-β-D-N-acetylglucosamine) has been shown for an N-acetylglucosaminidase from *Clostridium perfingens*	1
2	Carbohydrate-Binding Module Family 2	Modules of approx. 100 residues and which are found in a large number of bacterial enzymes. The cellulose-binding function has been demonstrated in many cases. Several of these modules have been shown to also bind chitin or xylan	1
3	Glycosyl Transferase Family 28	1,2-diacylglycerol 3-β-galactosyltransferase, 1,2-diacylglycerol 3-β-glucosyltransferase, UDP-GlcNAc: Und-PP-MurAc-pentapeptide β-N-acetylglucosaminyltransferase, Digalactosyldiacylglycerol synthase	1
4	Glycoside Hydrolase Family 10	Endo-1,4-β-xylanase, Endo-1,3-β-xylanase, Tomatinase, Xylan endotransglycosylase, Endo-β-1,4-glucanase, Arabinoxylan-specific endo-β-1,4-xylanase	1
5	Glycoside Hydrolase Family 120	β-xylosidase	1
6	Glycoside Hydrolase Family 51	Endoglucanase, Endo-β-1,4-xylanase, β-xylosidase, α-L-arabinofuranosidase, Cellobiohydrolase	1
7	Glycoside Hydrolase Family 43 / Subf 10	Xylan 1,4-β-xylosidase, α-L-arabinofuranosidase	1
8	Glycoside Hydrolase Family 39	α-L-iduronidase, β-xylosidase, α-L-arabinofuranosidase, β-glucosidase, β-galactosidase, Exo-β-1,4-glucanase / Cellodextrinase	1
9	Glycosyl Transferase Family 5	UDP-Glc: glycogen glucosyltransferase, ADP-Glc: starch glucosyltransferase, NDP-Glc: starch glucosyltransferase, UDP-Glc: α-1,3-glucan synthase, UDP-Glc: α-1,4-glucan synthase	1
10	Carbohydrate-Binding Module Family 4	Modules of approx. 150 residues found in bacterial enzymes. Binding of these modules has been demonstrated with xylan, β-1,3-glucan, β-1,3-1,4-glucan, β-1,6-glucan and amorphous cellulose but not with crystalline cellulose	1

11	Auxiliary Activity Family 7	Glucooligosaccharide oxidase, Chitooligosaccharide oxidase, Cellooligosaccharide dehydrogenase	1
12	Glycosyl Transferase Family 20	α,α-trehalose-phosphate synthase [UDP-forming], Glucosylglycerol-phosphate synthase, Trehalose-6-P phosphatise, GDP-valeniol: validamine 7-phosphate valeniolyltransferase	1
13	Glycoside Hydrolase Family 15	Glucoamylase, Glucodextranase, α,α-Trehalase, Dextran dextrinase	1
14	Glycosyl Transferase Family 4	Sucrose synthase, Sucrose-phosphate synthase, α-Glucosyltransferase, Lipopolysaccharide N-acetylglucosaminyltransferase, Phosphatidylinositol α-mannosyltransferase, GDP-Man: Man1GlcNAc2-PP-dolichol α-1,3-mannosyltransferase, GDP-Man: Man3GlcNAc2-PP-dolichol/Man4GlcNAc2-PP-dolichol α-1,2-mannosyltransferase, Digalactosyldiacylglycerol synthase, 1,2-diacylglycerol 3-glucosyltransferase, Diglucosyl diacylglycerol synthase, Trehalose phosphorylase, NDP-Glc: α-glucose α-glucosyltransferase / α,α-trehalose synthase, GDP-Man: Man2GlcNAc2-PP-dolichol α-1,6-mannosyltransferase, UDP-GlcNAc: 2-deoxystreptamine α-N-acetylglucosaminyltransferase, UDP-GlcNAc: ribostamycin α-N-acetylglucosaminyltransferase, UDP-Gal α-galactosyltransferase, UDP-Xyl α-xylosyltransferase, UDP-GlcA α-glucuronyltransferase, UDP-Glc α-glucosyltransferase, UDP-GalNAc: GalNAc-PP-Und α-1,3-N-acetylgalactosaminyltransferase, UDP-GalNAc: N,N'-diacetylbacillosaminyl-PP-Und α-1,3-N-acetylgalactosaminyltransferase, ADP-dependent α-maltose-1-phosphate synthase, UDP-GlcNAc: polypeptide α-N-acetylglucosaminyltransferase, UDP-GlcNAc: α-N-acetylglucosaminyltransferase	1
15	Glycosyl Transferase Family 1	UDP-glucuronosyltransferase, Zeatin O-β-xylosyltransferase, 2-Hydroxyacylsphingosine 1-β-galactosyltransferase, N-acylsphingosine galactosyltransferase, Flavonol 3-O-glucosyltransferase, Anthocyanidin 3-O-glucosyltransferase, Sinapate 1-glucosyltransferase, Indole-3-acetate β-glucosyltransferase, Flavonol L-rhamnosyltransferase, Sterol glucosyltransferase, UDP-Glc: 4-hydroxybenzoate 4-O-β-glucosyltransferase, Zeatin O-β-glucosyltransferase, Limonoid	1

		glucosyltransferase, UDP-GlcA: baicalein 7-O-β-glucuronosyltransferase, UDP-Glc: chalcone 4'-O-β-glucosyltransferase, Ecdysteroid UDP-glucosyltransferase, Salicylic acid β-glucosyltransferase, Anthocyanin 3-O-galactosyltransferase, Anthocyanin 5-O-glucosyltransferase, dTDP-β-2-deoxy-L-fucose: α-L-2-deoxyfucosyltransferase, UDP-β-L-rhamnose: α-L-rhamnosyltransferase, Zeaxanthin glucosyltransferase, UDP-Glc: flavone-6-C-glucosytransferase, UDP-Glc: cinnamate β-glucosyltransferase, UDP-Glc: hydroxycinnamic acid O-β-glucosyltransferase, UDP-Glc: cinnamoyl O-β-glucosyltransferase, UDP-Arap: flavone-C-arabinosytransferase, UDP-Glc: ginsenoside 3-O-glucosyltransferase, UDP-Glc: 3-O-glucosyl-protopanaxadiol-type ginsenoside 2''-O-glucosyltransferase	
16	Glycoside Hydrolase Family 13 / Subf 32	α-amylase, Maltotriose-producing α-amylase	1
17	Glycoside Hydrolase Family 149	β-1,3-glucan phosphorylase	1
18	Glycoside Hydrolase Family 81	Endo-β-1,3-glucanase	1
19	Glycoside Hydrolase Family 13 / Subf 20	Cyclic a-1,6-maltosyl-maltose hydrolase, α-glycosidase hydrolyzing pullulan, starch and γ-cyclodextrin, α-amylase, Maltogenic α-amylase, Neopullulanase, Pullulanase, Cyclomaltodextrinase	1
20	Polysaccharide Lyase Family 1 / Subf 5	Pectate lyase, Exo-polygalacturonate lyase	1
21	Glycoside Hydrolase Family 62	α-L-arabinofuranosidase	1
22	Glycoside Hydrolase Family 23	Lysozyme type G, Peptidoglycan lyase, also known in the literature as peptidoglycan lytic transglycosylase, Chitinase	1
23	Glycoside Hydrolase Family 109	α-N-acetylgalactosaminidase, β-N-acetylhexosaminidase	1
24	Glycoside Hydrolase Family 9	Endoglucanase, Endo-β-1,3(4)-glucanase / lichenase-laminarinase, Lichenase / endo-β-1,3-1,4-glucanase, Exo-β-1,4-glucanase / cellodextrinase, Cellobiohydrolase, Xyloglucan-specific endo-β-1,4-glucanase / endo-xyloglucanase, Exo-β-glucosaminidase, Endo-β-1,4-glucanase (Xanthanase)	1
25	Auxiliary Activity Family 1	Laccase / p-diphenol:oxygen oxidoreductase / ferroxidase, Ferroxidase, Laccase-like multicopper oxidase	1

26	Glycosyl Transferase Family 35	Glycogen or Starch phosphorylase	1
27	Glycoside Hydrolase Family 27	α-galactosidase, α-N-acetylgalactosaminidase, Isomalto-dextranase, β-L-arabinopyranosidase, Galactan:galactan galactosyltransferase	1
28	Glycoside Hydrolase Family 146	β-L-arabinofuranosidase	1
29	Glycoside Hydrolase Family 6	Endoglucanase, Cellobiohydrolase, Lichenase / Endo-β-1,3-1,4-glucanase	1
30	Carbohydrate Esterase Family 14	N-acetyl-1-D-myo-inosityl-2-amino-2-deoxy-α-D-glucopyranoside deacetylase, Diacetylchitobiose deacetylase, Mycothiol S-conjugate amidase	1

Supplementary Table S7. Tetra correlation among member species of *Cellulomonas* highlighted by a wide distribution of z-scores

**Organism name**	**Z-score**
Cellulomonas algicola TKZ-21	0.98478
Cellulomonas carbonis CGMCC 1.10786	0.98472
Cellulomonas fimi ATCC 484	0.98105
Cellulomonas biazotea NBRC12680	0.9798
Cellulomonas carbonis T26	0.97854
Cellulosimicrobium cellulans DSM 43879	0.97545
Cellulosimicrobium fucosivorans SE3	0.97504
Cellulosimicrobium terreum JCM 15619	0.97433
Cellulosimicrobium funkei JCM 14302	0.97422
Cellulomonas flavigena DSM 20109	0.97415
Cellulosimicrobium sp. MM	0.97404
Cellulomonas telluris CPCC 204705	0.97404
Cellulosimicrobium funkei NBRC 104118	0.9739
Cellulosimicrobium aquatile 3bp	0.97356
Sphaerisporangium cinnabarinum ATCC 31213	0.97345
Cellulosimicrobium cellulans NBRC 15516	0.97224
Cellulosimicrobium cellulans F16	0.97224
Cellulosimicrobium funkei U11	0.97186
Cellulomonas sp. B6	0.97166

Cellulosimicrobium composti BIT-GX5	0.97153
Cellulosimicrobium sp. I38E	0.97076
Cellulomonas cellasea DSM 20118	0.97074
Cellulomonas cellasea NBRC 3753	0.96996
Isoptericola variabilis 225	0.96962
Luteimicrobium subarcticum DSM 22413	0.96962
Flavimobilis soli DSM 21574	0.96769
Promicromonospora citrea JCM 3051	0.96679
Cellulomonas iranensis NBRC 101100 = JCM 18110	0.96679
Flavimobilis marinus DSM 19083	0.96534
Flavimobilis marinus CGMCC 1.3457	0.96516
Cellulomonas sp. Leaf334	0.96505
Cellulosimicrobium arenosum KCTC 49039	0.96384
Promicromonospora citrea ATCC 15908	0.96357
Cellulomonas massiliensis JC225	0.96308
Cellulomonas terrae NBRC 100819	0.9628
Cellulomonas rhizosphaerae NEAU-TCZ24	0.96136
Cellulomonas oligotrophica DSM 24482	0.96089
Cellulomonas uda NBRC 3747	0.96076
Actinotalea fermentans ATCC 43279	0.95947
Cellulomonas sp. Leaf395	0.95835
Cellulomonas sp. Root930	0.95813
Cellulomonas sp. Root485	0.95668
Cellulomonas composti NBRC 100758	0.95599

Cellulomonas xylanilytica NBRC 101102	0.95598
Cellulomonas sp. Root137	0.95586
Cellulomonas sp. A375-1	0.95555
Cellulomonas gelida JCM 1490	0.95385
Cellulomonas gelida NBRC 3748	0.95369
Cellulomonas persica NBRC 101101	0.95305
Actinotalea ferrariae CF5-4	0.95099
Cellulomonas humilata ATCC 25174	0.95011
Krasilnikoviella flava DSM 21481	0.94905
Cellulomonas soli DSM 24484	0.94622
Isoptericola cucumis CCM 8653	0.94581
Paraoerskovia marina DSM 21750	0.94541
Sanguibacter keddieii DSM 10542	0.94024
Sinomonas mesophila MPKL 26	0.93951
Aeromicrobium sp. Root472D3	0.9382
Oerskovia turbata JCM3160	0.93727
Oerskovia turbata NRRL B-8019	0.93723
Antribacter gilvus CFH 30434	0.9372
Miniimonas arenae KCTC 19750	0.93652
Cellulomonas marina CGMCC 4.6945	0.93642
Pseudoclavibacter chungangensis DSM 23821	0.93522
Sediminihabitans luteus NBRC 108568	0.93505
Sediminihabitans luteus DSM 25478	0.93502
Promicromonospora sukumoe DSM 44121	0.93435
Microbacterium album CGMCC 1.15794	0.93413
Promicromonospora soli CGMCC 4.7398	0.93403
Cellulomonas oligotrophica JCM 17534	0.93384

Curtobacterium citreum DSM 20528	0.93381
Aeromicrobium sp. Leaf272	0.93352
Pseudoclavibacter chungangensis DSM 23821	0.93331
Curtobacterium albidum DSM 20512	0.93319
Oerskovia jenensis DSM 46000	0.93279
Cellulomonas hominis DSM 9581	0.9324
Promicromonospora kroppenstedtii DSM 19349	0.93216
Curtobacterium citreum NS330	0.93164
Curtobacterium luteum DSM 20542	0.93128
Curtobacterium citreum JCM 1345	0.93112
Demequina salsinemoris NBRC 105323	0.93106
Cellulomonas hominis NBRC 16055	0.93052
Solirubrobacter pauli DSM 14954	0.93047
Salana multivorans DSM 13521	0.93034
Cellulomonas timonensis SN7	0.93009
Curtobacterium sp. ER1/6	0.92957
Curtobacterium luteum JCM 1480	0.92953
Curtobacterium luteum ATCC 15830	0.92951
Demequina rhizosphaerae NBRC 109397	0.92922
Sanguibacter gelidistatuariae ISLP-3	0.92876
Demequina maris NBRC 109392	0.92782
Demequina soli NBRC 109394	0.92753
Aeromicrobium sp. Leaf245	0.9272
Sanguibacter massiliensis Marseille-P3815	0.92703
Curtobacterium sp. MR_MD2014	0.92564
Puerhibacterium puerhi TLY-12	0.92528
Aeromicrobium massiliense JC14	0.92499
Clavibacter michiganensis subsp. capsici PF008	0.92479
Isoptericola jiangsuensis DSM 21863	0.92476
Curtobacterium luteum NS184	0.9247
